# *Tannerella forsythia* Tfo belongs to *Porphyromonas gingivalis* HmuY-like family of proteins but differs in heme-binding properties

**DOI:** 10.1042/BSR20181325

**Published:** 2018-10-23

**Authors:** Marcin Bielecki, Svetlana Antonyuk, Richard W. Strange, John W. Smalley, Paweł Mackiewicz, Michał Śmiga, Paulina Stępień, Mariusz Olczak, Teresa Olczak

**Affiliations:** 1 Faculty of Biotechnology, University of Wrocław, Wrocław, Poland; 2 Institute of Integrative Biology, University of Liverpool, Liverpool, U.K.; 3 School of Biological Sciences, University of Essex, Colchester, U.K.; 4 School of Dentistry, Institute of Clinical Sciences, University of Liverpool, Liverpool, U.K.

**Keywords:** HmuY, heme, Porphyromonas gingivalis, periodontitis, Tannerella forsythia, Tfo

## Abstract

*Porphyromonas gingivalis* is considered the principal etiologic agent and keystone pathogen of chronic periodontitis. As an auxotrophic bacterium, it must acquire heme to survive and multiply at the infection site. *P. gingivalis* HmuY is the first member of a novel family of hemophore-like proteins. Bacterial heme-binding proteins usually use histidine-methionine or histidine-tyrosine residues to ligate heme-iron, whereas *P. gingivalis* HmuY uses two histidine residues. We hypothesized that other ‘red complex’ members, i.e. *Tannerella forsythia* and *Treponema denticola* might utilize similar heme uptake mechanisms to the *P. gingivalis* HmuY. Comparative and phylogenetic analyses suggested differentiation of HmuY homologs and low conservation of heme-coordinating histidine residues present in HmuY. The homologs were subjected to duplication before divergence of *Bacteroidetes* lineages, which could facilitate evolution of functional diversification. We found that *T. denticola* does not code an HmuY homolog. *T. forsythia* protein, termed as Tfo, binds heme, but preferentially in the ferrous form, and sequesters heme from the albumin–heme complex under reducing conditions. In agreement with that, the 3D structure of Tfo differs from that of HmuY in the folding of heme-binding pocket, containing two methionine residues instead of two histidine residues coordinating heme in HmuY. Heme binding to apo-HmuY is accompanied by movement of the loop carrying the His^166^ residue, closing the heme-binding pocket. Molecular dynamics simulations (MD) demonstrated that this conformational change also occurs in Tfo. In conclusion, our findings suggest that HmuY-like family might comprise proteins subjected during evolution to significant diversification, resulting in different heme-binding properties.

## Introduction

Periodontal diseases belong to a group of infectious diseases, caused by an ecological shift in the composition of the subgingival biofilm, which results in inflammation and destruction of the tooth-supporting tissues [1,2]. The analysis of bacterial species isolated from subgingival samples has identified the relative abundance of the so-called ‘red complex’ members (*Porphyromonas gingivalis, Tannerella forsythia*, and *Treponema denticola*), which are associated with the clinical features of chronic periodontitis [3–5]. Amongst them, *P. gingivalis* is considered to be the main etiologic agent and keystone pathogen responsible for initiation and progression of chronic periodontitis [6,7].

*P. gingivalis* is a heme auxotroph, therefore it must acquire this compound to survive and cause efficient infection establishment. *In vivo*, free heme released from heme-containing proteins is not available because it is rapidly sequestered by host serum heme-scavenging proteins, which maintain the concentration of the free heme at very low levels [8]. However, heme may be derived from host serum albumin, hemopexin, and hemoglobin by *P. gingivalis* heme-binding proteins. Amongst well-characterized heme acquisition systems of *P. gingivalis* is that encoded by the *hmu* operon, comprising HmuR, a typical TonB-dependent receptor involved in heme transport across the outer membrane [9–12], HmuY, a heme-binding protein [13–15], and four proteins with unknown function. *P. gingivalis* HmuY binds Fe(III)- and Fe(II)protoporphyrin IX [13]. Characterization of the HmuY–heme complex has demonstrated that heme is in a low-spin Fe(III)/Fe(II), hexa-coordinate environment in the protein, with His^134^ and His^166^ acting as the heme ligands [14]. Our crystallographic studies have revealed a unique β-fold in the HmuY–heme protein structure and confirmed bis-histidine heme ligation [15].

Given the important role played by HmuY in the physiology and virulence of *P. gingivalis*, it is crucial to be able to reveal heme-binding mechanisms at the molecular level and to ascribe functions to the HmuY homologs of other ‘red complex’ members. The work presented here substantially extends our knowledge of *P. gingivalis* HmuY by presenting data on further structural characterization of this protein and novel data on a second member of *P. gingivalis* HmuY-like family, Tfo produced by *T. forsythia*.

## Experimental

### Bacterial strains and growth conditions

*P. gingivalis* A7436, *T. forsythia* ATCC 43037, and *Escherichia coli* ER2566 (New England Biolabs), Rosetta (DE3) (Novagen) strains were grown as described previously [16,17].

### Overexpression and purification of proteins

*P. gingivalis* A7436 HmuY protein (NCBI ID: CAM31898), lacking the signal peptide and first five amino acid residues (MKKIIFSALCALPLIVSLTSCGKKK) of the nascent secreted protein [15,18] and *T. forsythia* ATCC 43037 Tfo protein (NCBI ID: WP_046825712.1), lacking predicted signal peptide (MKMRNVMTLALVALSLAFVGC), were overexpressed and purified [17]. To construct expression plasmids containing the DNA sequences encoding appropriate proteins, pTriEx-4 vector (Novagen), respective primers and restriction enzymes were used as described previously [13,15,17,18]. For crystallization purposes of apo-HmuY, DNA sequence encoding HmuY protein, lacking 34 N-terminal amino acid residues was amplified using primers listed in Supplementary Table S1, digested with NcoI and XhoI and ligated into pTriEx-4 vector [13]. Concentrations of apo- and holo-HmuY were determined spectrophotometrically using the empirical molar absorption coefficients (ε_280_) 36.86 and 59.26 mM^−1^.cm^−1^, respectively [14]. The empirical molar absorption coefficient of Tfo (26.32 mM^−1^.cm^−1^) was calculated similarly.

### Protein–heme complex formation

Heme (hemin chloride; ICN Biomedicals) solutions and protein–heme complexes were prepared [14] and monitored in 100 mM Tris/HCl buffer, pH 7.5, containing 140 mM NaCl (TBS), or in 20 mM sodium phosphate buffer, pH 7.4, containing 140 mM NaCl (PBS) by recording UV-visible spectra with a single beam Ultrospec 2000 spectrophotometer (Biochrom Ltd.) or a double beam Jasco V-650 spectrophotometer (10 or 2 mm path length cuvettes, respectively). Titration curves were analyzed using equation for a one-site binding model and dissociation constant (*K*_d_) values were determined [19] using OriginPro 8 software (OriginPro Corporation). To analyze the redox properties of the iron present in the protein–heme complexes, sodium dithionite was used as the reductant and potassium ferricyanide as the oxidant [14,20].

### Circular dichroism and magnetic circular dichroism spectroscopies

Heme–protein complexes were prepared in 10 mM sodium phosphate buffer, pH 7.6. The protein concentration was adjusted to 10 μM (for far-UV CD), 100 μM (for CD in the visible region), or 40 μM (for magnetic CD spectroscopy (MCD) in the visible region). CD spectra were recorded at 200–260 nm (far-UV CD) or 340–660 nm (CD in the visible region) at 25°C using a Jasco J-715 or J-810 spectropolarimeter with a scan speed 50 nm min^−1^, response time 2 s, and a slit width of 1.0 nm. MCD spectra were recorded in the visible region at 25°C using a Jasco J-715 spectropolarimeter equipped with an electromagnet generating a magnetic field of 1.46 T, with a scan speed 200 nm min^−1^, response time 2 s, and a slit width of 1.0 nm. Measurements were made using a quartz cell with a 2-mm path length. Mean spectra were calculated from five independently recorded datasets.

### Native (PAGE), SDS/PAGE, and Western blotting

Samples were solubilized in appropriate application buffer at 37°C for 1 h or at 100°C for 5 min. For native PAGE, SDS was not included in the separating gel, and the sample solubilization was carried out in application buffer without SDS and DTT [21]. Gels were first stained for protein-bound heme with tetramethylbenzidine-H_2_O_2_ (TMB-H_2_O_2_) and counterstained for proteins with Coomassie Brilliant Blue R-250 or G-250 (CBB) [21,22]. Western blotting was carried out as described previously [17].

### Crystallization, X-ray data collection, processing and structure determination

Apo-Tfo protein was concentrated to 22 mg/ml, while apo-HmuY protein to 10 mg/ml. Crystals were grown using the hanging-drop method at room temperature by equilibration of 2 μl of the protein solution with 2 μl of reservoir solution, containing 3 M sodium malonate, pH 7.5, for apo-Tfo, or 2.4 M ammonium sulphate, 100 mM MES buffer, pH 6.0, for apo-HmuY. Crystals were flash frozen in liquid nitrogen using the reservoir solution as a cryoprotectant for apo-Tfo and 15% glycerol with reservoir solution for apo-HmuY. Crystals were stored in liquid nitrogen prior to data collection.

For apo-Tfo, X-ray data were collected at Diamond synchrotron U.K., beamline I04-1 at 100 K to 1.47 Å resolution and on the Rigaku X-ray generator FRE+ at the BARKLA X-ray Laboratory of Biophysics, University of Liverpool, at room temperature to 2.54 Å resolution. For apo-HmuY, X-ray data were collected at Diamond on beamline I03 at 100 K to 1.40 Å resolution. In-house X-ray data were processed and merged with HKL2000 [23], synchrotron data were processed with XDS [24] and merged by Aimless [25]. The room temperature Tfo structure was solved by molecular replacement using 3U22.PDB as the search model and then used as the starting model for the SR dataset. The structure of apo-HmuY was solved by molecular replacement using 3H8T.PDB as the search model. Both models were then refined using Refmac5 [26] and rebuilt in Coot [27]. Water molecules and ligands were added to both apo-Tfo and apo-HmuY models using Coot. Hydrogen atoms were added into the riding positions at the end of refinement. The quality of both models was assessed using MolProbity [28].

### Molecular dynamics simulations

Molecular dynamics simulations (MD) were performed using Gromacs 5.1.2 [29]. The crystal structures of apo-Tfo and holo-HmuY with the heme group removed were prepared for MD runs by adding hydrogen atoms and assigning charges to protein residues. Following energy minimization and solvation with TIP3P water and neutralization by adding sodium ions to the simulation box, the system was equilibrated under NVE ensemble for 100 ps and then switched to the NPT ensemble using the Parrinello–Rahman barostat [30], at a temperature of 300 K and 1 atm pressure. The system was then further equilibrated at 300 K for 300 ps. Production runs using the NPT ensemble with a time-step of 2 fs were then run for a total of 8 ns, by which time displacement of the ‘pocket loops’ were clearly established. The Particle-Mesh-Ewald (PME) sum method [31] was used for all electrostatic calculations with a cut-off distance of 1.0 nm. MD trajectories were examined using the VMD program [32].

### Susceptibility to proteolysis

HmuY and Tfo were subjected to trypsin digestion [15], as well as to digestion by proteases produced by both species. *P. gingivalis* cells were grown under high- or low-iron/heme conditions [13] and *T. forsythia* under high-iron/heme conditions [16,17] in the presence of added purified 1 μM HmuY or Tfo proteins. As controls, *P. gingivalis* or *T. forsythia* cultures without addition of the proteins was examined. Aliquots of samples were analyzed by SDS/PAGE and Western blotting [17].

### Heme sequestration experiments

Albumin–heme complex was prepared by incubating 120 μM stock solution of human albumin (Sigma; A-8763) with heme at a 1:0.9 protein to heme molar ratio to ensure that no free, uncomplexed heme remained in the preparation [22]. Human hemopexin (Sigma; H-9291) and bovine methemoglobin (MP Biomedicals; 151234) were also used. Co-incubation of apo-HmuY or apo-Tfo with hemoproteins and HmuY in apo-form with Tfo-Fe(III)heme complex was carried out in PBS (pH 7.6 and 6) at 37°C and monitored by UV-visible spectroscopy using holo-Tfo and apo-HmuY each at 10 μM [14,21].

### Bacterial cell fractionation

Portions of bacterial cultures were centrifuged at 20000×***g*** for 30 min at 4°C and supernatants filtered using sterile 0.22-μm filters (Roth) to separate the cell-free culture supernatant and cells. The cell pellets were washed twice with PBS and used to analyze the whole cell fraction. To separate outer membrane vesicles (OMVs), the filtered culture supernatant was centrifuged at 100000×***g*** for 2 h at 4°C using a Beckman fixed-angle rotor (Type 70 Ti), and pelleted membrane fractions were re-suspended in PBS. After ultracentrifugation, supernatant was concentrated 25× using Amicon Ultra-4 Centrifugal Filter Ultracel-10K units (Millipore).

### Quantitative reverse-transcriptase PCR

RNA was extracted from 0.5 × 10^8^ to 4 × 10^8^ cultured *P. gingivalis* or *T. forsythia* cells using the Total RNA Mini Kit (A&A Biotechnology). Purified RNA was treated with DNase I and purified using Clean-Up RNA Concentrator Kit (A&A Biotechnology). RNA integrity was verified by measuring absorbance and separating on agarose gel. Reverse transcription was carried out using 1 μg of RNA using SensiFAST cDNA Synthesis Kit (Bioline).

PCR was performed using SensiFAST SYBR No-ROX Kit (Bioline) and the LightCycler 96 System (Roche). Amplification reaction started with initial denaturation at 95°C for 2 min, 40 cycles of denaturation at 95°C for 5 s, primer annealing at 60°C for 10 s, and extension at 72°C for 20 s. The melting curves were analyzed to monitor the quality of PCR products. Relative quantitation of *tfo* and *hmuY* genes was determined in comparison with *16S rRNA* gene of *T. forsythia* (gene ID: L16495.1) and *P. gingivalis* (gene ID: 2552647) as references, using the ΔΔ*C*_t_ method. All samples were examined in triplicate for the target and reference genes. No template controls and negative controls were included as reported previously [33]. All primers used in the present study are listed in Supplementary Table S1.

The statistical analysis was performed using Student’s *t* test. Data were expressed as mean ± S.D. For statistical analysis, the GraphPad software (GraphPad Prism 5.0 Inc., San Diego, CA) was used.

### Collection of HmuY/Tfo homologs and phylogenetic analyses

Searches for homologs for *P. gingivalis* HmuY and *T. forsythia* Tfo in GenBank database were carried out using PSI-BLAST [34] assuming three iterations and *E*-value < 0.005. Next, the potential homologs containing domains annotated as HmuY, i.e. 316577, 213031, and 213030 were selected with *E*-value < 0.01 by searches of Conserved Domain Database [35] using rpsBLAST. The multiple sequence alignment was performed in MAFFT using accurate algorithm L-INS-i with 1000 cycles of iterative refinement [36]. The alignment was edited manually in JalView [37]. The set of 1292 amino acid sequences from various prokaryotic lineages was used to infer the global phylogenetic relationships between HmuY and Tfo homologs. In addition, all 369 sequences classified into Bacteroidia group were extracted and aligned using accurate algorithm T-Coffee combining sequence information with protein structures and profiles [38].

Phylogenetic trees were inferred using the Bayesian approach in MrBayes [39] and PhyloBayes [40], as well as maximum likelihood method in IQ-TREE [41] and morePhyML [42] based on PhyML [43].

In MrBayes analyses, we applied mixed+I+Γ(5) models and two independent runs, each using 72 or 8 Markov chains (for the global and Bacteroidia sets, respectively). The trees were sampled every 100 generations for 20000000 generations. In the final analysis, we selected the last 110421 to 45431 trees, that reached the stationary phase and convergence.

In the PhyloBayes analysis, the convergence was reached only for the Bacteroidia set. Therefore, results only for this set were presented. In this case, the WAG+Γ(5) model was applied as proposed in ProtTest [44]. Two independent Markov chains were run for 100000 generations with one tree sampled for each generation. The last 25000 trees from each chain were collected to compute posterior consensus trees.

The tree calculated with (more)PhyML was based on the LG+Γ(5) or WAG+I+Γ(5) models, as found in ProtTest, for the global and Bacteroidia sets, respectively. We applied the best search algorithm NNI+SPR. In IQ-TREE, we used LG+R8 or WAG+R7 models for the global and Bacteroidia sets, respectively, as suggested by ModelFinder [45]. To assess significance of branches, we performed a non-parametric bootstrap analysis on 100 or 1000 replicates and the approximate likelihood ratio test (aLRT) [46] assuming 1000 or 10000 replicates in IQ-TREE, for the global and Bacteroidia sets, respectively.

### Accession numbers

The structures of apo-HmuY (PDB ID: 6EWM) and apo-Tfo (PDB ID: 6EU8) were deposited at http://www.rcsb.org/structure/6EWM and http://www.rcsb.org/structure/6EU8, respectively.

## Results

### Phylogenetic analysis of HmuY and Tfo homologs

Database searches resulted in 3540 potential distant homologs to *P. gingivalis* HmuY and *T. forsythia* Tfo. Despite a quite large divergence, it was possible to align the sequences, select conserved regions and infer phylogenetic relationships (Supplementary Figures S1 and S2). Majority of HmuY and Tfo homologs belong to phylum Bacteroidetes and related phyla. We also found homologs in many Spirochetes but not in *T. denticola*.

Eleven main groups can be recognized in the phylogenetic tree (Supplementary Figure S1). *P. gingivalis* HmuY and *T. forsythia* Tfo are placed within the G3 clade amongst other Bacteroidia sequences, which are also distributed into G1 and G5. Other clades include representatives of other bacterial groups. However, sequences assigned to a given phylum or class, e.g. those from Bacteroidetes, are not always clustered together but are separated into different clades. Such distribution suggests that ancestral *hmuY* genes were subjected to duplications before divergence of the main Bacteroidetes lineages. Alternatively, horizontal gene transfers could occur between these lineages. More clear horizontal gene transfers probably occurred between members of different phyla.

**Figure 1:**
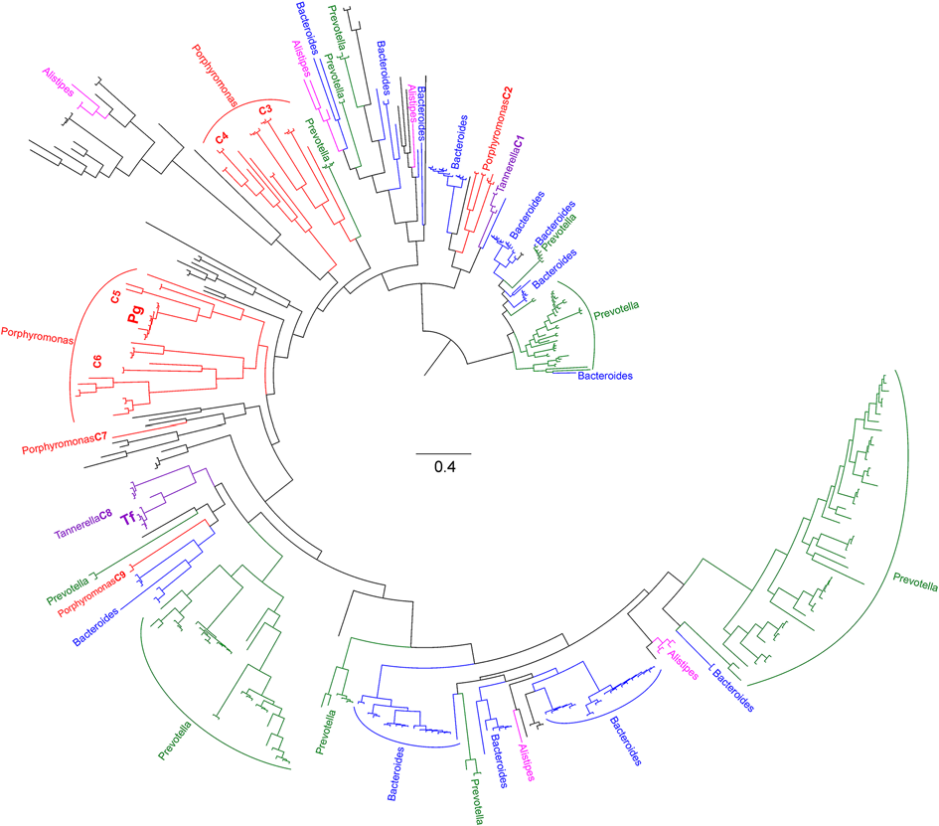
The phylogenetic tree obtained in MrBayes for the HmuY homologs in Bacteroidia Lineages of main genera are marked in different colors. Nine clusters including *Porphyromonas* and *Tannerella* are indicated. *P. gingivalis* HmuY (Pg) and *T. forsythia* Tfo (Tf) are shown in bold. The full tree with support values obtained by various methods is presented in Supplementary Figure S3.

*P. gingivalis* HmuY and *T. forsythia* Tfo are separated into different clades of class Bacteroidia and are not close homologs (Figure 1 and Supplementary Figure S3). Many sequences from *Porphyromonas* do not form a monophyletic clade either but are distributed into six clades separated by other genera from the Bacteroidia. *Tannerella* sequences are also separated into two unrelated clades. Such distribution implies that the genes encoding the HmuY and Tfo homologs were duplicated before differentiation of Bacteroidia into the current genera and various copies were maintained in the individual lineages.

### Organization of *hmu* operons

**Figure 2:**
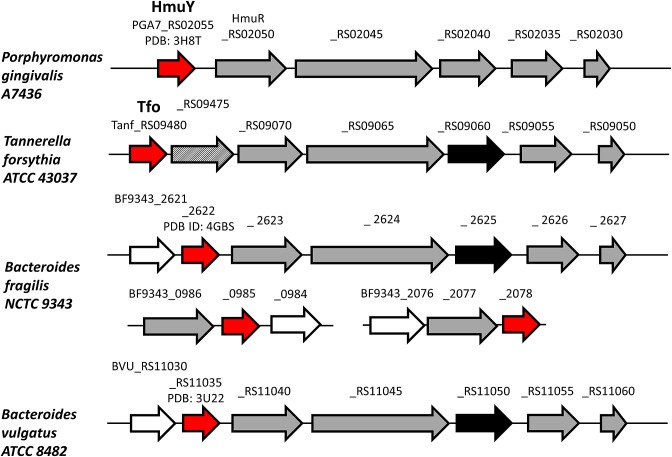
Schematic presentation of genes encoding HmuY homologs *P. gingivalis* HmuY and its homologs in *T. forsythia* (Tfo), *Bacteroides fragilis* and *Bacteroides vulgatus* (shown in red) and other genes identified in the *P. gingivalis hmu* and *hmu*-like operons (shown in gray) are shown. The gene marked in black does not exhibit homology to the gene encoding PGA7_RS02040 (putative ATPase) in *P. gingivalis.* Genes marked with open arrows do not exist in all bacteria presented here. The gene marked in gray diagonal stripes encodes a TonB-dependent outer-membrane receptor, which is less homologous to *P. gingivalis* HmuR.

Genetic organization and amino acid sequences of products of respective genes located in the *hmu* operon, which is a potential counterpart of a typical *hem* operon found in other bacteria [47,48], are different in *P. gingivalis* and other Bacteroidetes members (Figure 2). Interestingly, the *T. forsythia hmu*-like operon possesses an additional HmuR homolog. The first TonB-dependent outer-membrane receptor in this operon (Tanf_RS09475) is less similar to the *P. gingivalis* HmuR (PGA7_RS02050) (Supplementary Figure S4) because it lacks two His residues engaged in heme coordination in HmuR and other typical heme TonB-dependent outer-membrane receptors [11]. Instead, Tyr and Met residues are present in homologous positions and their close neighborhood. The second gene encoding HmuR homolog in this operon (Tanf_RS09470) is very similar to the *P. gingivalis* HmuR (Supplementary Figure S4), suggesting the possibility of a heme transport function.

### Sequence conservation of HmuY and Tfo homologs

Conserved regions from HmuY homologs are distributed along the whole sequence but do not include two His residues coordinating heme in *P. gingivalis* HmuY (Supplementary Figure S5). However, it is possible to find other residues conserved in all or majority of sequence phylogenetic groups, which are probably involved in formation of structures or folds, which apparently did not change during evolution. On the other hand, each or only few groups have the residues that are uniquely conserved only in them, which may be important in some functional or structural differentiation of proteins in individual groups of homologs.

**Figure 3:**
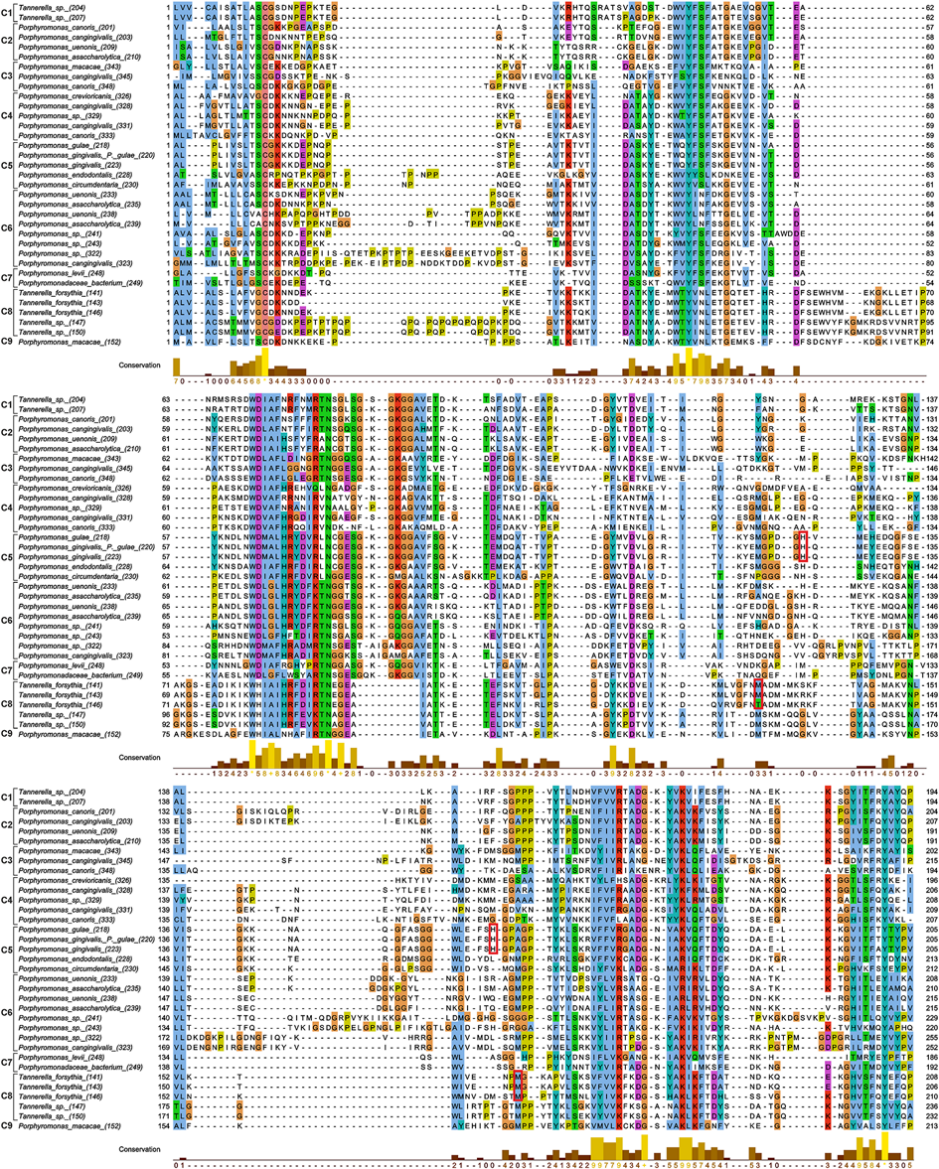
Amino acid sequence alignment of *Porphyromonas* and *Tannerella* sequences that are homologous to HmuY and Tfo Nine clusters indicated in Figure 1 are shown to the left of sequence names. Histidine and methionine residues coordinating heme in *P. gingivalis* HmuY and *T. forsythia* Tfo, respectively, are marked by red boxes.

**Figure 4:**
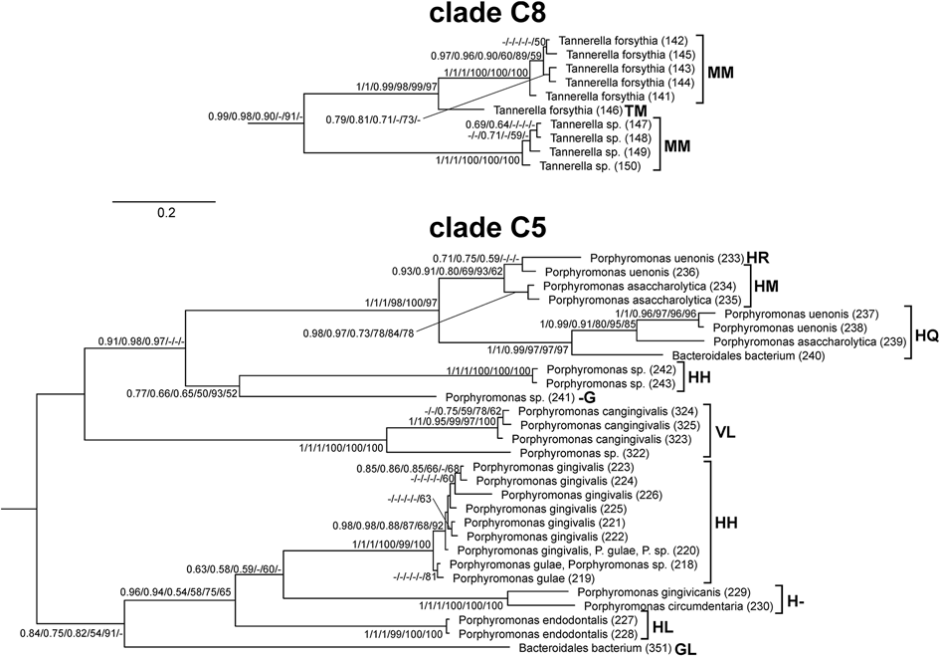
Extracted clades C5 and C8 including the closest homologs to *P. gingivalis* HmuY and *T. forsythia* Tfo, respectively Amino acid residues aligned at the homologous positions to the HmuY histidine residues coordinating heme are shown at sequence names in clade C5, whereas residues corresponding to Tfo methionine residues are shown in clade C8. The values at nodes indicate in the following order: posterior probabilities found in MrBayes and PhyloBayes as well as support values calculated by aLRT based on a Shimodaira–Hasegawa-like procedure and non-parametric bootstrap calculated both in (more)PhyML and IQ-TREE. The posterior probabilities <0.5 and the percentages < 50% are omitted or indicated by a dash ‘-’.

Since the two His residues coordinating heme in *P. gingivalis* HmuY were not conserved across large evolutionary distances, we focussed on the homologs from the *Porphyromonas* and *Tannerella* to study the conservation in homologs within the same genera. The His residues found in *P. gingivalis* HmuY are generally poorly conserved in the majority of sequences (Figure 3). However, there are some sequences which preserve these residues in the homologous positions. Besides *P. gingivalis* (placed in clade C5), the first His residue is also found in other *Porphyromonas* species (Figure 4), whereas the second His residue is much less conserved. Nevertheless, besides His, other residues (Met, Cys, Tyr, Lys) can also serve as axial ligands to the heme iron [49]. Met or Lys were found in the homologous position to the first His in sequences from the distantly related C2, C3, and C7 clades, whereas Met or Lys homologous to the second His are present in the representatives of clades C3, C4, and C6.

Besides *T. forsythia* sequence (WP_060827954.1, 146) (Figure 3), other representatives of this genus do not have residues appropriate for heme binding in the positions homologous to the His residues. However, in the distance of only six alignment sites from the first His and three sites from the second His, there are Met residues, being good candidates for heme coordination. The first Met (Figure 3) is present in almost all *Tannerella* of clade C8 and also various *Porphyromonas* sequences distributed into clades C4, C5, C6, and C9 (Figure 4). The sequences from *Tannerella* belonging to clade C1 has Tyr in the homologous position similar to three *Porphyromonas* sequences. Interestingly, *Porphyromonas* sp. (WP_044125400.1, 241) has a His residue in this position. The second Met is conserved in all *Tannerella* in clade C8 and in some *Porphyromonas* sequences in clades C3, C5, and C6. The sequence from *P. levii* in the C7 clade has a His residue in the vicinity of this site, which can be easily aligned with the Met residues. These results suggest that in the neighborhood of HmuY histidines there are a number of potential residues that can coordinate heme in many *Porphyromonas* and *Tannerella*. The distribution of the considered residues in sequences grouped in the phylogenetic tree (Figure 4) implies that the His and Met residues could be also present in ancestors of the C5 and C8 clades.

### Tfo binds heme but in a manner different from HmuY

**Figure 5:**
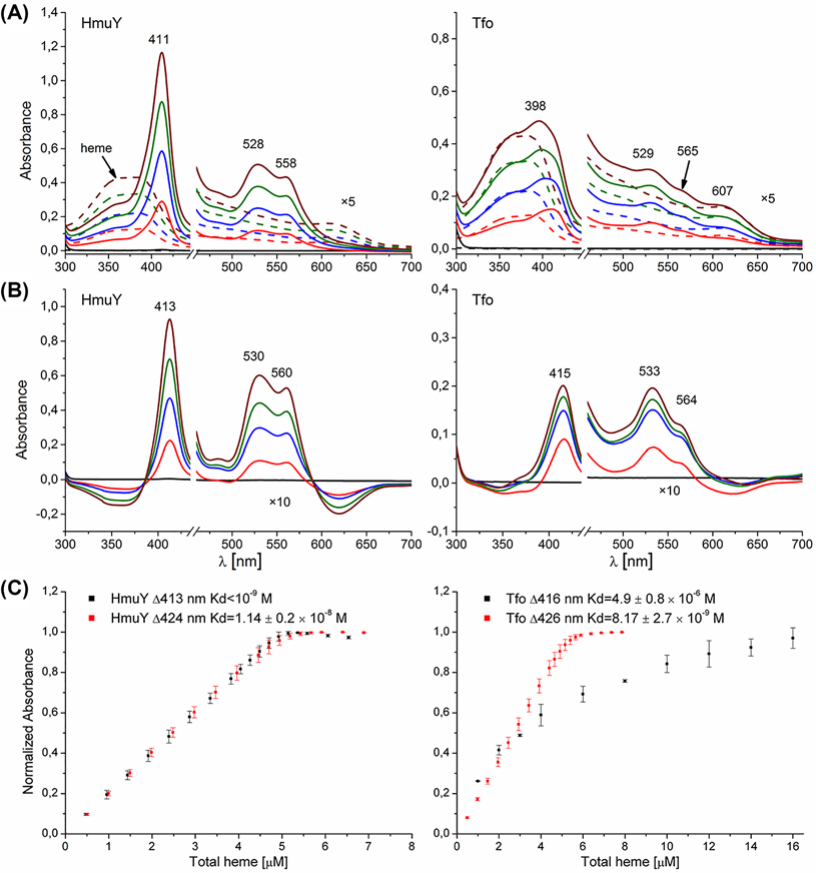
Heme titration experiments of *P. gingivalis* HmuY and *T. forsythia* Tfo UV-visible absorption (**A**) and difference (**B**) spectra of HmuY and Tfo recorded after titration of proteins (10 μM) with heme are shown. (**C**) The curves were generated after titration of 5 μM protein samples with heme by measuring the difference spectra between the protein+heme and heme-only samples. Samples were examined under air (oxidizing) conditions (black) or reduced by sodium dithionite (red). Results are shown as mean ± S.D. from three independent experiments.

The comparative analyses of HmuY homologs suggest that not only the HmuY hemophore-like protein of *P. gingivalis*, but also *T. forsythia* Tfo may be engaged in heme acquisition. Compared with HmuY, which exhibited a Soret λ_max_ in the 411 nm region [14], the Soret maximum determined for Tfo was at 398 nm (Figure 5A). In addition, compared with the HmuY Q band maxima at 528 and 558 nm [14], those for Tfo were located at 529, 565, and 607 nm. These results were corroborated by difference spectrum analysis, although slightly different values of absorbance maxima were observed (Figure 5B).

**Figure 6:**
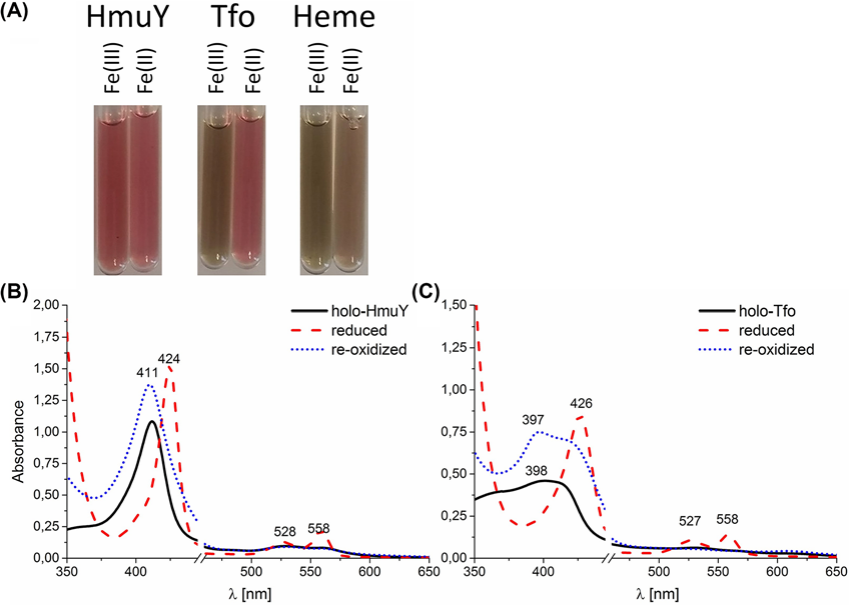
Analysis of heme binding to purified *P. gingivalis* HmuY and *T. forsythia* Tfo under different redox conditions examined by UV-visible spectroscopy (**A**) Colors of 150 μM HmuY and Tfo proteins complexed with heme (protein:heme ratio 1:1) in PBS are shown. (**B,C**) UV-visible absorption spectra of HmuY–heme and Tfo–heme complexes are presented. Samples (10 μM proteins) in complex with heme were examined under air (oxidizing) conditions and subsequently reduced by sodium dithionite, and re-oxidized by potassium ferricyanide.

Compared with the HmuY protein, which after overexpression, purification, and concentration existed under air (oxidizing) conditions in solution as red-colored complex, Tfo gave a green-color, which was visible as a brown-colored complex after heme titration (Figure 6A). After reduction, the Tfo-heme solution gave a red-colored complex, highly similar to HmuY–heme solution (Figure 6A). The Soret peak maximum of Tfo red shifted and a single peak emerged at 426 nm, compared with 424 nm for HmuY (Figure 6B). Moreover, reduction produced increased and well-resolved Q bands at wavelengths almost identical with those observed for HmuY, suggesting a hexa-coordinate low-spin Fe(II)heme in Tfo. The heme bound to Tfo was further re-oxidized, resulting in the Soret band shift back to 397 nm, compared with 411 for HmuY.

**Figure 7:**
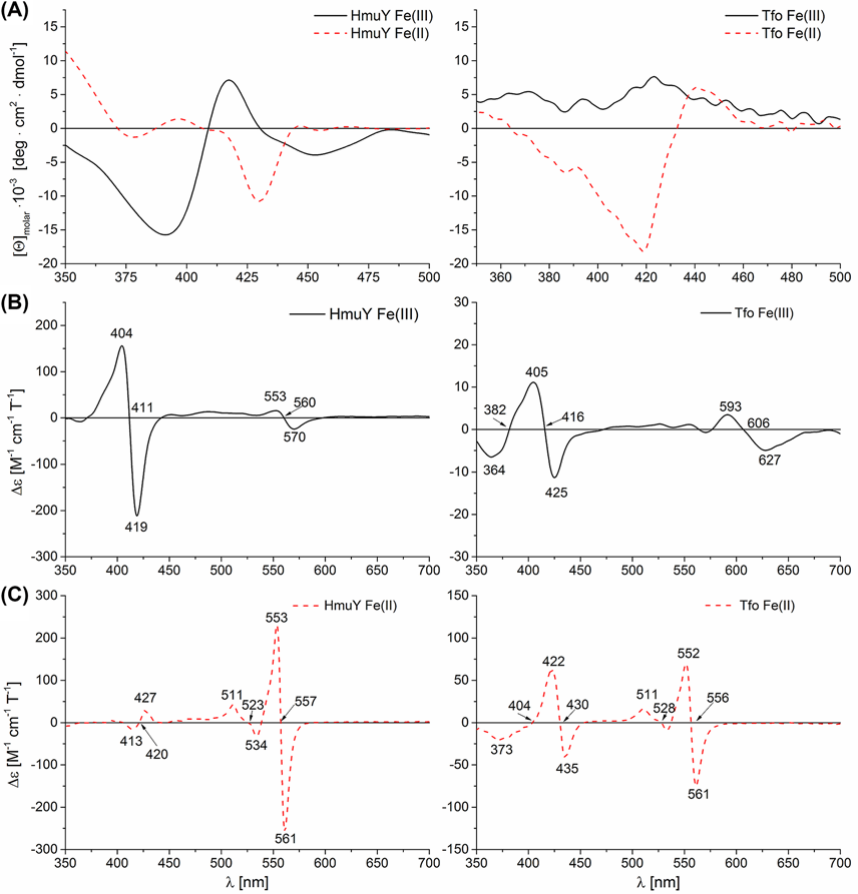
Analysis of heme binding to purified *P. gingivalis* HmuY and *T. forsythia* Tfo under different redox conditions Heme binding was monitored in the visible region by CD (**A**) and MCD (**B**,**C**) spectroscopies. Samples were examined under air (oxidizing) conditions (A,B) and subsequently reduced by sodium dithionite (A,C).

The CD spectra of Tfo determined in the visible region under oxidizing conditions differed from those observed for HmuY (Figure 7A). The main feature was the lack of a negative Cotton effect in the ferric heme form. Reduction in Tfo resulted in the minimum similar to HmuY (Figure 7A). Further, the resulting MCD spectra (Figure 7B,C) were compared with data obtained for other hemoproteins. However, based on our results we were unable to find an accurate match for the heme coordination chemistry in Tfo–Fe(III)heme complex. In contrast, the spectra recorded under reducing conditions are similar to those observed in hemoproteins with hexa-coordinate, low-spin heme.

Previously, using UV-visible spectroscopy with a single beam spectrophotometer [19,50], we estimated the *K*_d_ of heme binding under oxidizing conditions to the HmuY protein to be approximately 0.25 × 10^−8^ M. Here we carried out a more thorough analysis using a double-beam instrument and found that HmuY binds heme with *K*_d_ value < 10^−9^ M (Figure 5C). This tendency was preserved under reducing conditions (Figure 5C). Compared with HmuY, Tfo-bound heme with lower ability under oxidizing conditions. However, reduction resulted in significantly higher heme-binding ability to Tfo, comparable or even slightly higher with that observed for HmuY. Similar to HmuY, heme binding to Tfo did not cause significant changes in the secondary structure of the protein, as determined by far-UV CD analysis (data not shown).

### Tfo exists as monomers and is more susceptible to proteolysis than HmuY

In contrast with HmuY, which was shown to form a dimer even after SDS/PAGE, Tfo usually migrated during electrophoresis as a single band (Supplementary Figure S6A). Cross-linking studies revealed a similar pattern observed for HmuY and Tfo (Supplementary Figure S6A). Size-exclusion chromatography showed that both apo- and holo-Tfo existed under oxidizing conditions in a monomeric form, whereas holo-HmuY exhibited a tendency to form, in part, a dimer (Supplementary Figure S6B). Under reducing conditions, only holo-Tfo migrated, in part, as a dimer.

**Figure 8:**
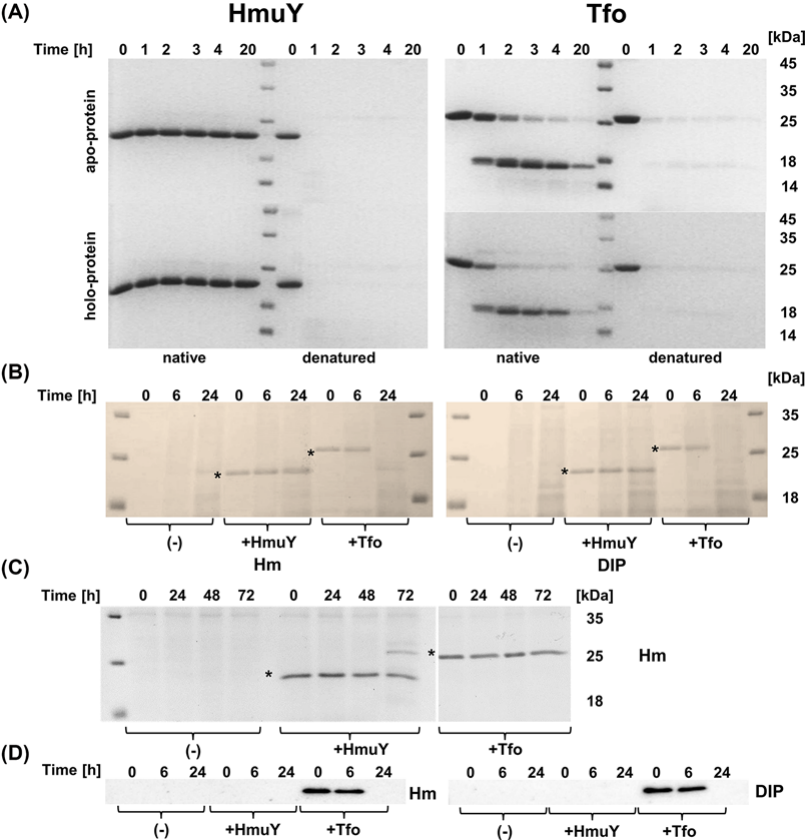
Proteolytic susceptibility of *P. gingivalis* HmuY and *T. forsythia* Tfo (**A**) Both proteins in apo- and holo-forms in their native states (native) and after thermal denaturation (denatured) were subjected to trypsin digestion and visualized by staining with CBB G-250. Susceptibility of proteins to *P. gingivalis* (**B**) or *T. forsythia* (**C**) proteases was examined by growing bacterial cells under high- (Hm) or low-iron/heme (DIP) conditions in the presence of the purified HmuY or Tfo proteins (marked with asterisks). Protein samples collected at indicated time points were separated by SDS/PAGE and visualized by staining with CBB G-250 (B). (**D**) The presence of Tfo in *P. gingivalis* cultures was also examined by Western blotting using rabbit polyclonal anti-Tfo antibodies.

Previously, we demonstrated that *P. gingivalis* HmuY is completely resistant to several proteases [15,22,51]. In contrast with those findings, we showed that Tfo was more susceptible to trypsin digestion (Figure 8A). Our observations were further corroborated by experiments demonstrating *P. gingivalis* or *T. forsythia* growth in the presence of added purified HmuY or Tfo proteins. In contrast with HmuY, which was completely resistant during *P. gingivalis* growth, Tfo was digested by *P. gingivalis* proteases, whereas proteases produced by *T. forsythia* were not able to digest both proteins (Figure 8B–D).

### Tfo sequesters heme from albumin–heme complex under reducing conditions

**Figure 9:**
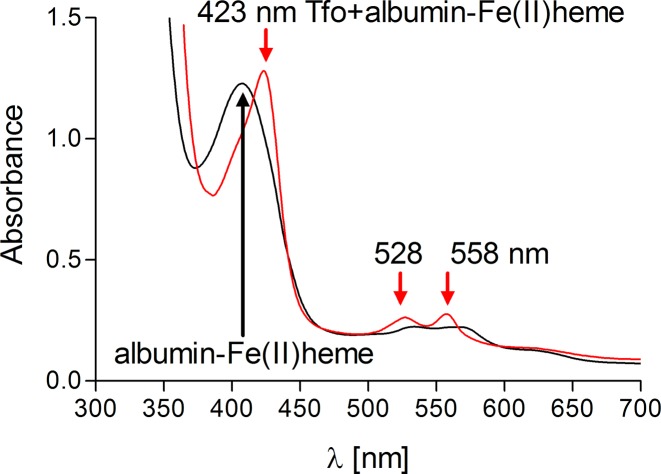
Sequestration of heme by *T. forsythia* Tfo from human albumin complexed with Fe(II)heme Samples were examined by UV-visible spectroscopy under reducing conditions formed by addition of sodium dithionite.

One of the hypothesized functions of Tfo, similar to *P. gingivalis* HmuY, would be to gain heme in an environment where heme levels are tightly restricted by host heme-sequestering proteins. Our studies clearly demonstrated that *P. gingivalis* HmuY efficiently extracted heme from methemoglobin, albumin–heme [21,22], and hemopexin–heme complexes (Supplementary Figure S7). Here we found that Tfo was not able to sequester heme present in methemoglobin or bound to albumin or hemopexin when in the Fe(III)heme form (data not shown). However, we observed that Tfo extracted heme from the albumin–heme complex but only under reducing conditions (Figure 9), although complexation of Fe(II)heme bound to hemopexin was not demonstrated (data not shown). To analyze possible syntrophy between *P. gingivalis* and *T. forsythia*, we examined the interactions between apo-HmuY and holo-form of Tfo and showed that HmuY efficiently sequestered Fe(III)heme which had been complexed to Tfo (Supplementary Figure S8).

### 3D structure of apo-HmuY

**Figure 10:**
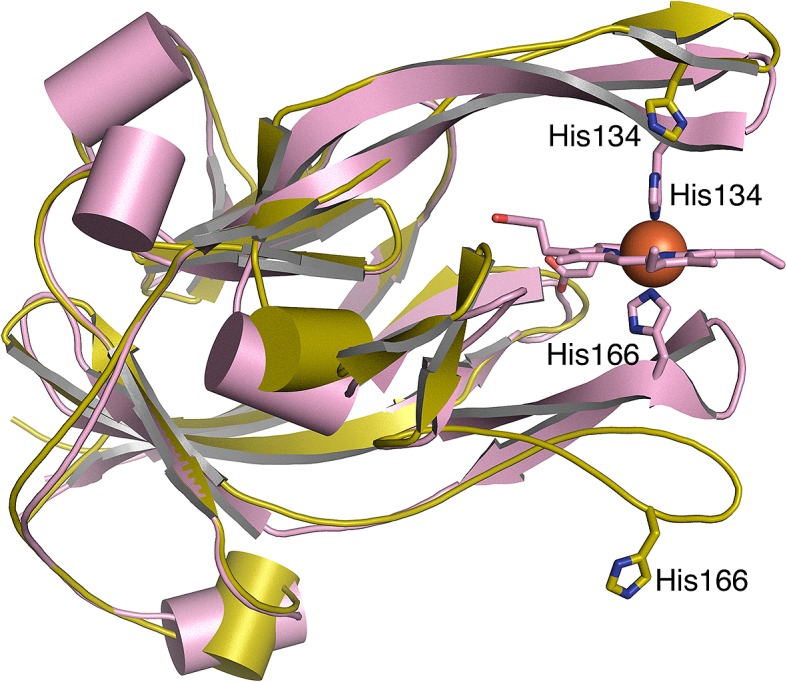
The structures of the *P. gingivalis* holo- and apo-HmuY monomers In the apo-protein (yellow), the loop containing the axial heme ligand His^166^ is no longer constrained by binding to the heme group and adopts a new position, rotating and reorienting the His^166^ side chain externally toward the surface of the protein. This results in an opening up of the heme-binding pocket compared with the holo-protein (pink; PDB ID: 3H8T). A smaller shift away from the heme-binding site occurs for the β-strand containing the axial ligand His^134^. This part of the structure is more rigid. The rms difference in the two aligned structures is 1.8 Å, calculated for 179 residues using the cealign command in PyMol (The PyMOL Molecular Graphics System, version 1.8 Schrödinger, LLC).

To further characterize *P. gingivalis* HmuY, we successfully solved its 3D structure in apo-form. The structure of apo-HmuY (PDB ID: 6EWM) was determined by molecular replacement starting from the holo-HmuY model (PDB ID: 3H8T) (Supplementary Table S2). Compared with holo-HmuY [15], one can see the opening up of the heme-binding pocket in the apo-form (Figure 10). The loop containing the axial heme ligand, His^166^, has moved significantly and the His^166^ side chain faces the surface of the apo-protein (the His166NE2 atom is shifted from the heme by approximately 16 Å). This loop must close up when the heme group enters the pocket. The opening up of the heme-binding pocket observed in the crystal structure of apo-protein (Figure 10) was reproduced by MD (Supplementary Figure S9).

### 3D structure of Tfo

**Figure 11:**
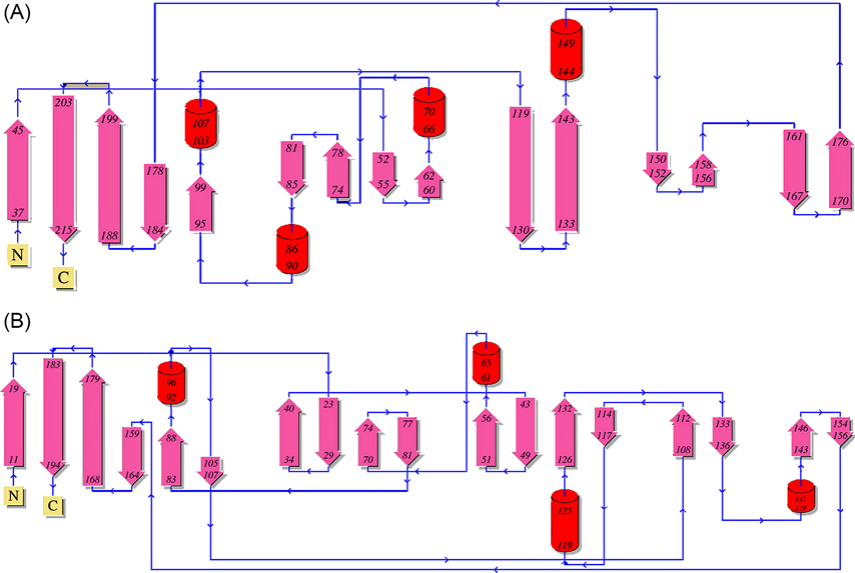
Topological schemes for *P. gingivalis* HmuY and *T. forsythia* Tfo Comparison of *P. gingivalis* HmuY (**A**) and *T. forsythia* Tfo (**B**) showed that the main difference lies in the region of the structure known to bind the heme moiety in HmuY.

**Figure 12:**
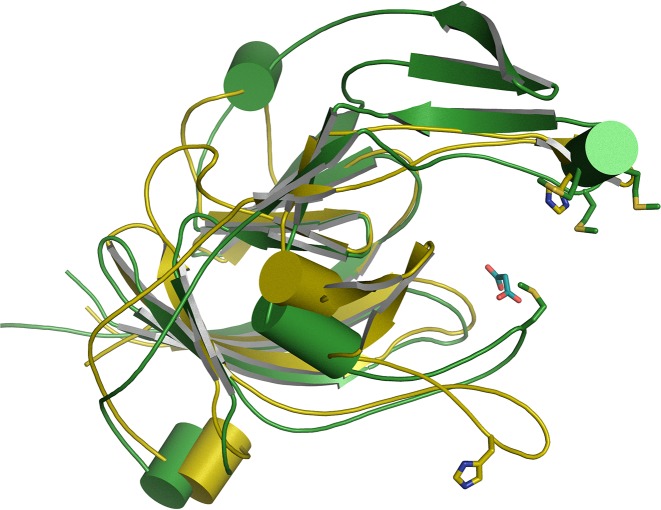
Cartoon representations of the aligned structures of apo-HmuY (yellow) and apo-Tfo (green) monomers A malonate molecule from the crystallization medium is present in the heme-binding pocket of apo-Tfo. The two His residues that bind as axial ligands to the heme group in holo-HmuY, and the Met residues occupying equivalent structural locations in Tfo, are shown as sticks. The rms difference in the two aligned structures is 3.5 Å, calculated for 160 residues using the cealign command in PyMol (The PyMOL Molecular Graphics System, version 1.8 Schrödinger, LLC).

**Figure 13:**
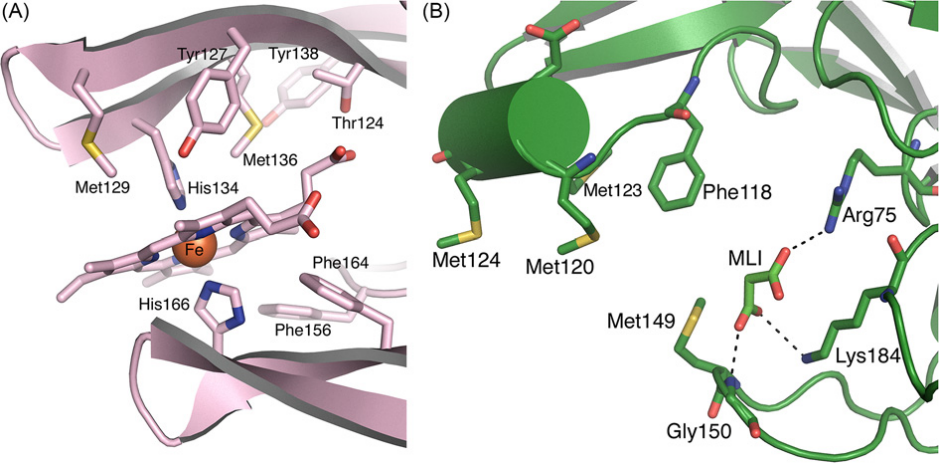
Close-up of the heme-binding pocket in *P. gingivalis* holo-HmuY (**A**) and the equivalent site in *T. forsythia* apo-Tfo (**B**) The Tfo site lacks the His residues that are the axial ligands to the Fe-heme in HmuY. The binding pocket is narrower in the apo-Tfo crystal structure, with a malonate ligand (MLI) and the Met^149^ in positions occupied by the heme in HmuY. The malonate forms H-bonds (2.8-3.1 Å, indicated by dashed lines) to residues Gly^150^, Arg^75^, and Lys^184^ and to water molecules (not shown). Numbering of amino acid residues in the case of Tfo is shown according to the protein lacking the signal peptide sequence (20 amino acid residues), which was crystallized and examined by X-ray analysis.

**Figure 14:**
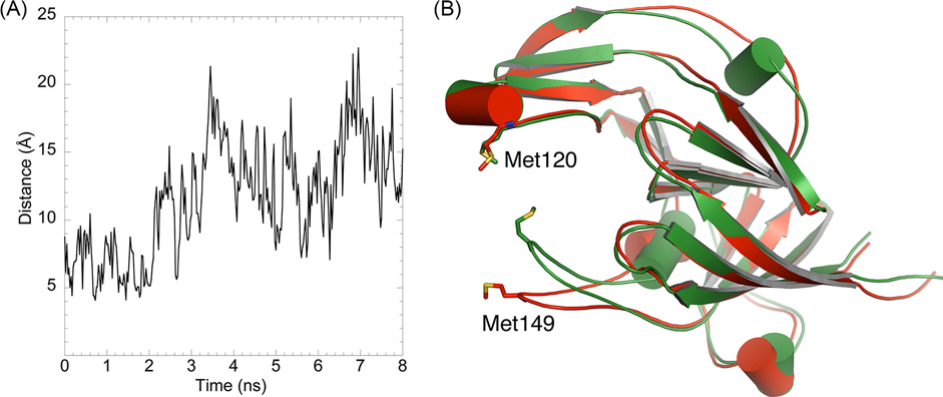
Molecular dynamics of *T. forsythia* apo-Tfo (**A**) shows the variation in the distance between Met^120^ and Met^149^ SD atoms during 8 ns of an all-atom MD, beginning with the equilibrated crystal structure data. (**B**) shows the initial structure (green) and the simulated structure after 8 ns (red). The opening up of the potential heme-binding pocket by approximately 10 Å is largely due to the movement of the loop containing Met^149^. The rms difference between the initial and MD simulated structures is 2.2 Å. Numbering of amino acid residues in the case of Tfo is shown according to the protein lacking the signal peptide sequence (20 amino acid residues), which was crystallized and examined by X-ray analysis.

The structure of apo-Tfo (PDB ID: 6EU8) was determined in space group P32_1_ to a resolution of 1.47 Å, with two molecules in the asymmetric unit and final R/R-free of 18.4/21.9% (Supplementary Table S2). Analysis of Tfo structure using the PDBePISA server [52] suggested that the two molecules do not form a biologically significant dimer. The topology of the primarily β-sheet secondary structure of the protein shows a series of antiparallel β-strands in five groups. Similar to apo-HmuY (Figure 11A), apo-Tfo exhibited topology typical of all-β strand structure, with the main distinction apparent in the fold forming the heme-binding pocket, where two long β-strands in HmuY that form one side of the pocket are replaced in Tfo by four shorter β-strands (Figure 11B). Figures 12 and 13 show the HmuY heme-binding pocket and the corresponding region in Tfo. The binding pocket in the apo-Tfo crystal structure was occupied by a malonate molecule from the crystallization medium, which was bound by hydrogen bonds to Gly^150^, Arg^75^, and Lys^184^ residues (numbering of amino acid residues according to the protein lacking the signal peptide sequence, crystallized, and examined by X-ray analysis). Similar residues were involved in the binding of an unknown ligand to the HmuY-like heme-binding protein from *Bacteroides vulgatus* ATCC 8482 (BVU_2192; PDB ID: 3U22). The hydrogen bonding between malonate and these residues restricted movement of the loops covering the binding pocket, resulting in the closed conformation. In contrast, the homologous protein from *Bacteroides fragilis* (BF9343_2622; PDB ID: 4GBS) did not possess such a bound ligand. All-atom MD, similar in procedure to those validated using HmuY, showed that enlargement of the pocket opening is possible in the absence of malonate. The resulting open conformation would presumably then allow for the heme group to be accommodated in the pocket in holo-Tfo (Figure 14). Tfo lacks the two His residues that are required for axial ligation of the heme in HmuY. Instead, Met, Lys, and Tyr residues are potentially available for binding the heme. Based on spectroscopic studies, we assumed that two methionine residues are the best candidates. This assumption was also made based on the phylogenetic analyses and comparison of the HmuY and Tfo 3D structures. However, to date we do not have structure of the holo-Tfo, as 1.8 Å data collected from brown holo-Tfo crystals only reveal presence of the apo-form of the protein. Anaerobic crystallization and protein storage may be needed to preserve heme in the binding pocket.

### *T. forsythia* expresses Tfo under low-iron/heme conditions

**Figure 15:**
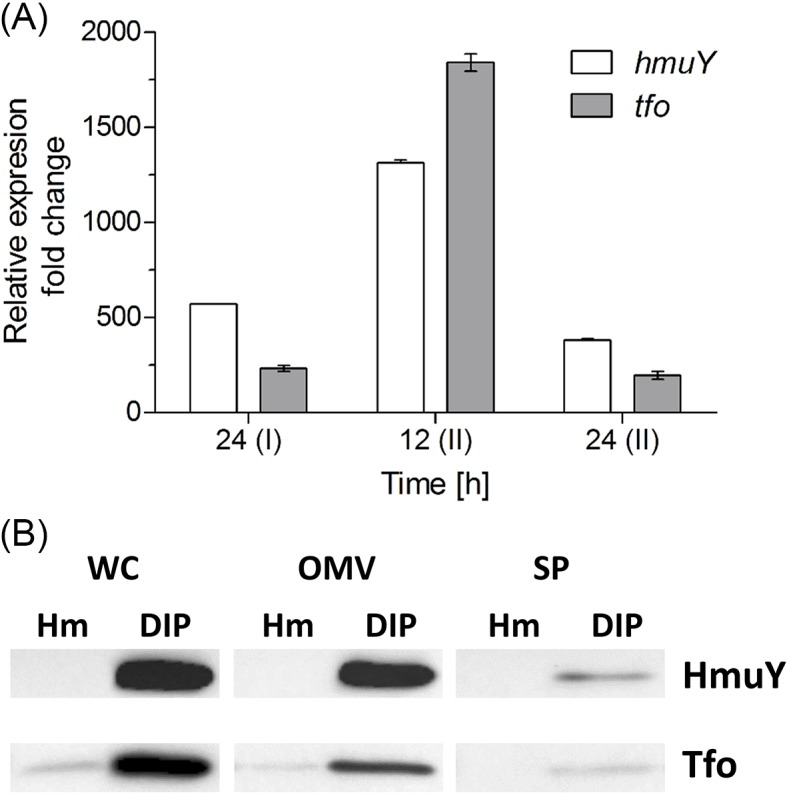
Expression of *P. gingivalis* HmuY and *T. forsythia* Tfo during bacterial growth (**A**) Relative changes in levels of transcripts in bacteria grown under low- compared with high-iron/heme conditions at indicated time points at the first (I) and second (II) passage as determined by RT-qPCR are shown. (**B**) Bacteria were grown in liquid culture media under high- (Hm) or low-iron/heme (DIP) conditions for 24 h and both whole cultures and bacterial cells were subjected to centrifugation and/or ultracentrifugation. To enable visualization of the soluble HmuY and Tfo by immunoblotting, the culture media were concentrated 25× by ultrafiltration. Abbreviations: OMV, outer membrane vesicles; SP, soluble protein shed from bacterial outer membrane; WC, whole bacterial cell.

The HmuY protein is associated with both the bacterial outer membrane and OMVs through a lipid anchor [18,53], and can also be shed as an intact, soluble protein as a result of the limited proteolytic processing by *P. gingivalis* lysine-specific gingipain K (Kgp) [15,18]. *P. gingivalis* produces higher levels of HmuY when the bacterium grows under low-iron/heme conditions or as a biofilm constituent [13,18], as well as intracellularly in host cells [54]. Similar to *hmuY* mRNA and HmuY protein, both *tfo* transcript (Figure 15A) and Tfo protein (Figure 15B) were produced at higher levels in bacteria grown under low-iron/heme conditions, as compared with high-iron/heme conditions. Distribution pattern of the Tfo protein between whole *T. forsythia* cells, OMVs, and culture medium containing soluble protein shed from the outer membrane was also similar, as compared with *P. gingivalis* HmuY (Figure 15B).

## Discussion

*P. gingivalis* and *T. forsythia* have been indicated as species prevalent in consortia within subgingival pockets associated with chronic periodontitis [4,5]. *P. gingivalis* surface-exposed proteins, proteins associated with OMVs, or secreted proteins are crucial for its own virulence and effective invasion of host cells [2,53–56]. Moreover, *P. gingivalis* virulence factors are important for adherence to and aggregation with other oral bacteria, including *T. forsythia* [57–58], which in turn exhibits a growth-promoting effect toward *P. gingivalis* [59]. All these findings suggest the synergistic virulence potential and ecological relationship of *P. gingivalis* and *T. forsythia* [55,57–60]. We hypothesize that other oral bacteria, especially those classified as the ‘red complex’, might utilize similar proteins to acquire heme and increase their virulence. Compared with *E. coli*, which predominantly expresses porins in the outer membrane to acquire nutrients [61], members of Bacteroidetes phylum possess a different outer membrane architecture, rich in lipoproteins. Amongst them are *P. gingivalis* HmuY (PGA7_RS02055), three *B. fragilis* NCTC 9343 (BF9343_0985, BF9343_2078, BF9343_2622), and one *B. vulgatus* (BVU_RS11035) potential HmuY homologs, as well as *T. forsythia* Tfo (Tanf_RS09480). The majority of lipoproteins are cell surface-exposed and could be released into the environment, including *P. gingivalis* HmuY [18,53], *T. forsythia* Tfo [62,63], and *B. fragilis* HmuY homologs [64]. In addition, many genes encoding lipoproteins are adjacent to genes encoding TonB-dependent outer membrane receptors and likely promote the nutrient acquisition, including internalization of heme.

Our previous work on *P. gingivalis* extensively characterized a novel, unique *hmu* heme acquisition mechanism, requiring the HmuY protein. We demonstrated that *P. gingivalis* HmuY not only binds free heme, but can wrest heme from methemoglobin directly [21] and thus functions similar to typical secreted hemophores, which are engaged in heme transfer from the host hemoproteins to the outer membrane receptors [65,66]. HmuY is also able to compete with albumin [21], which is the normal front-line heme scavenger *in vivo*, as well as acquiring heme from serum hemopexin (the present study). Importantly, we demonstrated here that heme may also be sequestered under air conditions by HmuY from Tfo–heme complex. We suggest that heme bound to Tfo might represent a heme reservoir for *P. gingivalis*, which could be accessed by the action of HmuY during phases of colonization when *T. forsythia* dominates over *P. gingivalis*.

To shed more light on the heme-binding mechanism displayed by *P. gingivalis* HmuY, we successfully crystallized the protein in apo-form. We experimentally verified that heme binding was accompanied by a movement of the loop carrying the His^166^ residue. As compared with apo-HmuY, analysis of the 3D structure of apo-Tfo confirmed differences revealed by spectroscopic analyses, mainly in the fold forming the heme-binding pocket and the lack of two His residues coordinating heme in HmuY. Our spectroscopic data did not exclude the possibility that Met residues might coordinate heme. Importantly, they demonstrated that heme can be bound to Tfo efficiently under reducing conditions, which favors coordination through Met residues. Although the apo-Tfo structure exhibited a ‘closed’ form, partly caused by malonate binding, MD showed that it could open up to accommodate the heme. Comparison of apo-HmuY with apo-Tfo showed the same kind of opening of this region and it is the ‘same’ loop, containing Met^149^ (Met^169^ according to the numbering of amino acid residues in the full length protein) that moves significantly compared with the crystal structure of the closed conformation of apo-Tfo. It has been shown that classical hemophores from *Serratia marcescens* [67,68] and *Pseudomonas aeruginosa* [69–71] also exhibit structural rearrangements upon heme binding to His^32^ and Tyr^75^, which allows for both heme pick up and subsequent release for transfer to the outer membrane heme-binding receptors. However, a recent study demonstrated that His^32^ is not conserved in all secreted hemophores. For example, HasA from *Yersinia pestis* and *Yersinia pseudotuberculosis* coordinate heme by employing a single Tyr^75^, and that the structures of the proteins in both apo- and holo-forms are quite similar [72–74]. Analogously, we suspected that Tfo might use only one Met residue to coordinate heme. However, our data suggested that Tfo might coordinate Fe(II)heme through Met^120^ and Met^149^ residues (Met^140^ and Met^169^ according to numbering of amino acid residues in the full length protein). An example of bacterial protein coordinating heme using two Met residues, Met^66^ and Met^153^, is the *Streptococcus pyogenes* surface protein (Shp), which transfers heme to HtsA, the lipoprotein component of HtsABC (ABC transporter of Gram-positive bacteria) [75]. In contrast with Shp, which binds heme under both oxidizing and reducing conditions, Tfo requires reducing conditions for efficient heme binding. Similar to Shp [76], Tfo does not sequester heme from methemoglobin.

It is not surprising that progression of chronic periodontitis correlates with the formation of periodontal pockets, which is associated with decrease in oxidation-reduction potential (Eh) and a reduced environment preferred by anaerobic bacteria [77,78]. Since *P. gingivalis* and *T. forsythia* reside in periodontal pockets as components of a ‘red complex’, one would expect that they would both require similar conditions for growth. However, it is worth noting that the early periodontal pathogenesis in the gingival pocket is characterized by reducing conditions but not yet typified by bleeding. Under these conditions the main heme source will be albumin before hemoglobin enters from bleeds. If *T. forsythia* can flourish in this early environment, when *P. gingivalis* is not yet dominant, then any heme capture from albumin (reduced) becomes a more important part of a bacterial pool for future access by other hemophore-like proteins, such as HmuY. It is noteworthy that the affinity of albumin for Fe(II)heme is lower than for heme in the Fe(III) state [79,80] and that this may facilitate heme capture by Tfo. Moreover, reduced conditions influence properties of iron coordination to methionine more significantly than to histidine, thus allowing efficient heme binding also to Tfo and heme sequestration from the albumin-Fe(II)heme complex by Tfo. This effect could be explained by bis-Met heme ligation in Tfo, which results in stabilization of the reduced state as compared with bis-His ligation in HmuY [81]. As compared with bis-His ligation employing nitrogen atoms, stabilization of the reduced state of the protein by bis-Met ligation occurs since coordination of two methionyl sulphur atoms, serving as good electron acceptors, results in rise of the redox potential [82]. Based on the theory of hard and soft acids and bases [83,84], one may hypothesize that under oxidizing conditions bis-Met ligand binding is destabilized. In this context, HmuY may be important for effective heme acquisition in a heme-limited environment, especially in a polymicrobial plaque biofilm community and within host cells, especially under air conditions.

In conclusion, our data presented here on further characterization of HmuY as well as new findings on *T. forsythia* Tfo not only shed more light on the molecular bases of the novel mechanism of heme uptake of ‘red complex’ members but also add to characterization of proteins composing this novel family of hemophore-like proteins.

## Supporting information

**Supplementary Figure S1:** The phylogenetic tree obtained in MrBayes for the HmuY homologs from all phyla. Main bacterial lineages are marked in different colors. Eleven main groups can be recognized in the phylogenetic tree. Three big groups (G1, G3 and G5) are dominated by Bacteroidetes sequences representing various classes of this phylum: Bacteroidia, Chitinophagia, Cytophagia, Flavobacteriia, Saprospiria and Sphingobacteriia. *P. gingivalis* HmuY and *T. forsythia* Tfo are placed within the G3 clade among other Bacteroidia sequences. Groups G9 and G10 include almost exclusively Proteobacteria representatives and are placed close to group G11 including Euryarchaeota sequences and one α-proteobacterium. A monophyletic group is G7, which contains representatives of Spirochaetes. Potential horizontal gene transfers occurred likely from Euryarchaeota to a Rhizobiales bacterium (G11), from d-Proteobacteria to two Spirochaeta species (G8), from Bacteroidetes to cyanobacterium *Lyngbya confervoides* (G6) and cyanobacterium *Hassallia byssoidea* (G5) as well as from Bacteroidetes to a Verrucomicrobia bacterium, an Euryarchaeota archaeon and a γ-proteobacterium (G1). In these cases one or few sequences from one phylum are significantly clustered within many sequences of the other distantly related phylum. The full tree with support values obtained by various methods is presented in **Supplementary Figure S2**.

**Supplementary Figure S2:** The full phylogenetic tree (cladogram) obtained in MrBayes for the HmuY homologs from all phyla. Main bacterial lineages are marked in different colors. The values at nodes indicate in the following order: posterior probabilities found in MrBayes as well as support values calculated by approximate likelihood-ratio test (aLRT) based on a Shimodaira-Hasegawa-like procedure and non-parametric bootstrap calculated both in (more)PhyML and IQ-TREE. The posterior probabilities < 0.5 and the percentages < 50% are omitted or indicated by a dash “-“.

**Supplementary Figure S3:** The full phylogenetic tree (cladogram) obtained in MrBayes for the HmuY homologs in Bacteroidia. Main Bacteroidia classes are marked in different colors. The values at nodes indicate in the following order: posterior probabilities found in MrBayes and PhyloBayes as well as support values calculated by approximate likelihood-ratio test (aLRT) based on a Shimodaira-Hasegawa-like procedure and non-parametric bootstrap calculated both in (more)PhyML and IQ-TREE. The posterior probabilities < 0.5 and the percentages < 50% are omitted or indicated by a dash “-“.

**Supplementary Figure S4:** Amino-acid sequence alignment of TonB-dependent outermembrane receptors. Proteins encoded in *P. gingivalis* hmu (HmuR_A7436) and *T. forsythia hmu*-like (Tf_HmuR_I and Tf_HmuR_II) operons are shown. Conserved histidine (H), glutamic acid (E) residues and FRAP, NPNL motifs present in classical TonB-dependent receptors transporting heme are marked in red and blue, respectively.

**Supplementary Table S1:** Primers used in this study.

**Supplementary Table S2:** Data collection and refinement statistics.

## Abbreviations

CD: circular dichroism spectroscopy
MCD: magnetic CD
MD: molecular dynamics simulation
OMV: outer membrane vesicles
PBS: 20 mM sodium phosphate buffer, pH 7.4, containing 140 mM NaCl
