# Supplementary material for: Tannerella forsythia Tfo belongs to Porphyromonas gingivalis HmuY-like family of proteins but differs in heme-binding properties
Source: Biosci Rep. 2018 Oct 23;38(5):BSR20181325. doi: 10.1042/BSR20181325 (PMC6200708; doi:10.1042/BSR20181325)
Supplement: Supplementary file 1 [file bsr20181325_Supp1.pdf]

Supplementary material

Tannerella forsythia Tfo belongs to *Porphyromonas gingivalis* HmuY-like family of proteins but differs in heme-binding properties

Marcin Bielecki[1], Svetlana Antonyuk[2], Richard W. Strange[3], John W. Smalley[4], Paweł Mackiewicz[1], Michał Śmiga[1], Paulina Stępień[1], Mariusz Olczak[1], Teresa Olczak[1]*

[1]Faculty of Biotechnology, University of Wrocław, Wrocław, Poland
[2]Institute of Integrative Biology, University of Liverpool, Liverpool, UK
[3]School of Biological Sciences, University of Essex, Colchester, UK
[4]School of Dentistry, Institute of Clinical Sciences, University of Liverpool, Liverpool, UK

***Corresponding author:** Teresa Olczak, Laboratory of Medical Biology, Faculty of Biotechnology, University of Wrocław, 14A F. Joliot-Curie St., 50-383 Wrocław, Poland; teresa.olczak@uwr.edu.pl; Tel. (+48) 71 3752 612; Fax (+48) 71 3757 661.

Supplementary Table S1 Primers used in this study.

Name	DNA sequence (5’→3’)	Product length (bp)	Locus ID, gene abbreviation	Description
F_qTfo<br>R_qTfo	GGATGCGACAAGAAAGACGA<br>CCTTTGGCTGGGATGGTTTC	182	LN624459, *tfo*	Amplify fragment of *T. forsythia tfo* gene (qRT-PCR)
F_16sRNA_Tf<br>R_16sRNA_Tf	AGTCGCGTGAAGGATGACTG<br>CTTAACAGCCCACCTACGCA	195	NC_016610, *16S rRNA*	Amplify fragment of *T. forsythia 16S rRNA* gene (qRT-PCR)
HYq4_F<br>HYq4_R	GCTTCGAAATACGAAACGTG<br>TATATCCGTCTGTCGGAACG	119	PGA7_RS02055, *hmuY*	Amplify fragment of *P. gingivalis hmuY* gene (qRT-PCR) (Gmiterek *et al*., 2013)
16SrRNA-F<br>16SrRNA-R	CTTGAGTTCAGTGGCGGCAG<br>AGGGAAGACGGTTTTCACCA	378	PGA7_RS09290, *16S rRNA*	Amplify fragment of *P. gingivalis 16S rRNA* gene (qRT-PCR) (Gmiterek *et al*., 2013)
FHmuY_EAV<br>RHmuY_EAV	CGTCCATGGAAGCAGTAACC AAAACCGTAAC<br>AGTCTCGAGTTATTTAACGGG GTATGTATAAGTGAAAG	546	PGA7_RS02055, *hmuY*	Amplify DNA sequence encoding HmuY protein lacking 34 N-terminal amino-acid residues

Sequences recognized and cleaved by restriction enzymes are underlined.

Gmiterek, A., Wojtowicz, H., Mackiewicz, P., Radwan-Oczko, M., Kantorowicz, M., Chomyszyn-Gajewska, M., Frąszczak M, Bielecki M, Olczak M, Olczak T (2013) The unique *hmuY* gene sequence as a specific marker of *Porphyromonas gingivalis* infection. *PLoS One* 8(7):e67719

Supplementary Table S2 Data collection and refinement statistics.

	Apo-HmuY	Apo-Tfo	Apo-Tfo-r
**Data collection**			
Temperature	100	100	273
Source	IO3 Diamond	IO2 Diamond	BARKLA
Space group	$P2_1$	$P3_121$	$P3_121$
Cell dimensions			
a, *b*, *c* (Å)	59.33, 42.95, 95.08	68.05, 68.05, 90.22	68.30 68.30 94.07
α, β, γ (°)	90, 107.87, 90	90, 90, 120	90, 90, 120
Resolution (Å)	90.9-1.40 (1.42-1.40)*	49-1.47 (1.5-1.47)*	30-2.55 (2.64-2.55)*
R_{sym} or R_{merge}[a]	0.014 (0.520)	0.033 (2.1)	0.072 (0.96)
R_{pim}	0.01 (0.43)	0.011 (0.76)	0.028 (0.42)
$I / \sigma I$	5.9 (2.5)	30.2 (1.2)	24.0 (2.1)
CChalf	0.98 (0.68)	1 (0.6)	
Completeness (%)	100.0 (74.0)	100 (99.9)	100 (99.8)
Redundancy	4.3 (3.5)	9.4 (9.4)	7.3 (5.9)
**Refinement**			
Resolution (Å)	45.2 4 - 1.40	49.34 - 1.47	
No. reflections	82229	39662	
R_{work} / R_{free}[b]	0.131 / 0.171	0.184 / 0.219	
No. atoms			
Protein	2980	1566	
Water	748	236	
SO4 ions	15		
Glycerol	30		
B-factors			
Protein	13.5	31.1	
Water	31.4	40.5	
R.m.s. deviations			
Bond lengths (Å)	0.015	0.012	
Bond angles (°)	1.656	1.502	
PDB code	6EWM	6EU8	

[a] $R_{merge} = \sum|I_i - I_m|/\sum I_i$, where I_i is the intensity of the measured reflection and I_m is the mean intensity of all symmetry related reflections.

[b] $R_{cryst} = \Sigma||F_{obs}| - |F_{calc}||/\Sigma|F_{obs}|$, where F_{obs} and F_{calc} are observed and calculated structure factors.

$R_{free} = \Sigma_T||F_{obs}| - |F_{calc}||/\Sigma_T|F_{obs}|$, where T is a test data set of about 5% of the total reflections randomly chosen and set aside prior to refinement.

*Numbers in parentheses represent the value for the highest resolution shell.

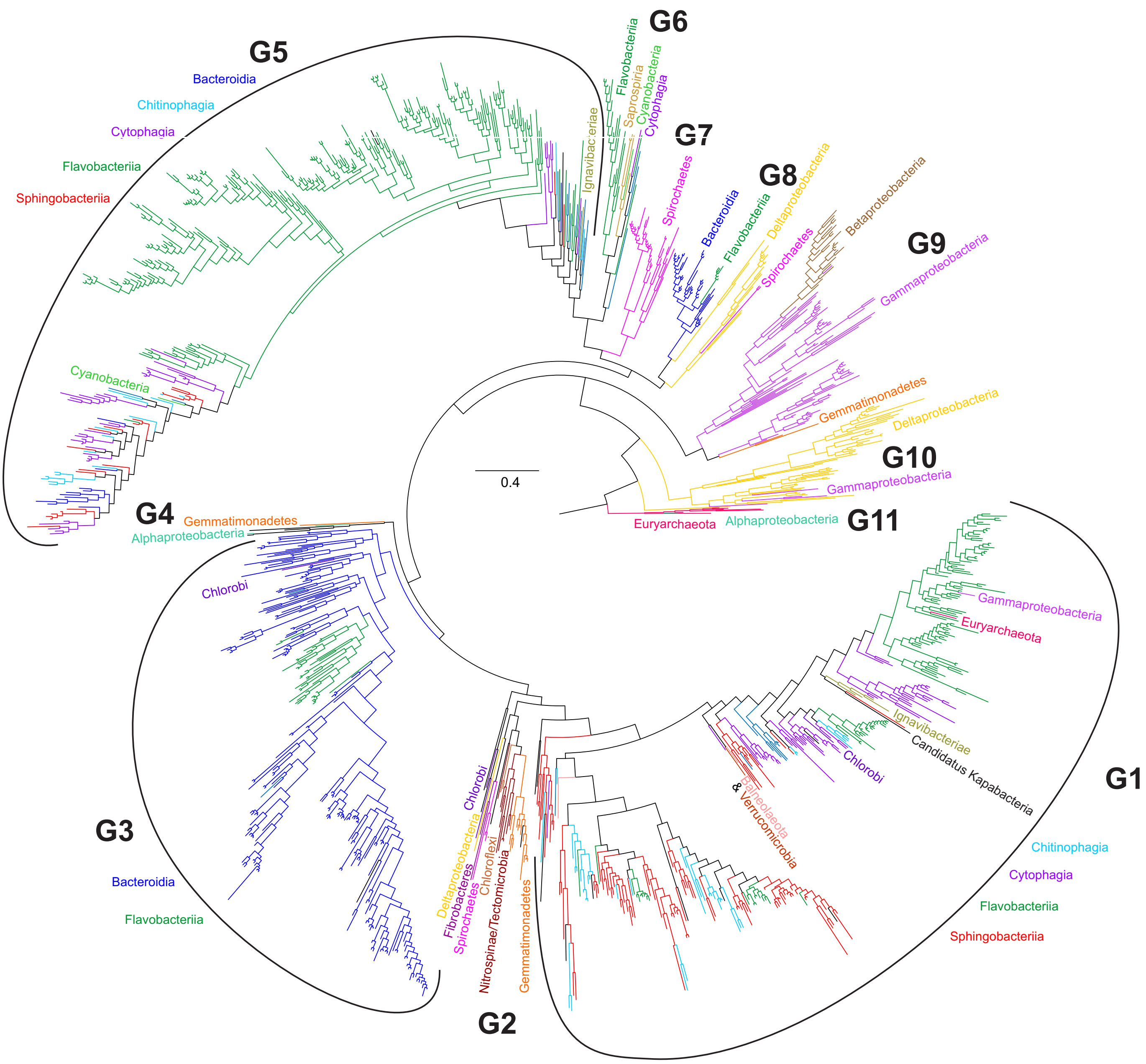

Supplementary Figure S1 The phylogenetic tree obtained in MrBayes for the HmuY homologs from all phyla. Main bacterial lineages are marked in different colors. Eleven main groups can be recognized in the phylogenetic tree. Three big groups (G1, G3 and G5) are dominated by Bacteroidetes sequences representing various classes of this phylum: Bacteroidia, Chitinophagia, Cytophagia, Flavobacteriia, Saprospiria and Sphingobacteriia. *P. gingivalis* HmuY and *T. forsythia* Tfo are placed within the G3 clade among other Bacteroidia sequences. Groups G9 and G10 include almost exclusively Proteobacteria representatives and are placed close to group G11 including Euryarchaeota sequences and one α-proteobacterium. A monophyletic group is G7, which contains representatives of Spirochaetes. Potential horizontal gene transfers occurred likely from Euryarchaeota to a Rhizobiales bacterium (G11), from δ-Proteobacteria to two Spirochaeta species (G8), from Bacteroidetes to cyanobacterium *Lyngbya confervoides* (G6) and cyanobacterium *Hassallia byssoidea* (G5) as well as from Bacteroidetes to a Verrucomicrobia bacterium, an Euryarchaeota archaeon and a γ-proteobacterium (G1). In these cases one or few sequences from one phylum are significantly clustered within many sequences of the other distantly related phylum. The full tree with support values obtained by various methods is presented in **Supplementary Figure S2**.

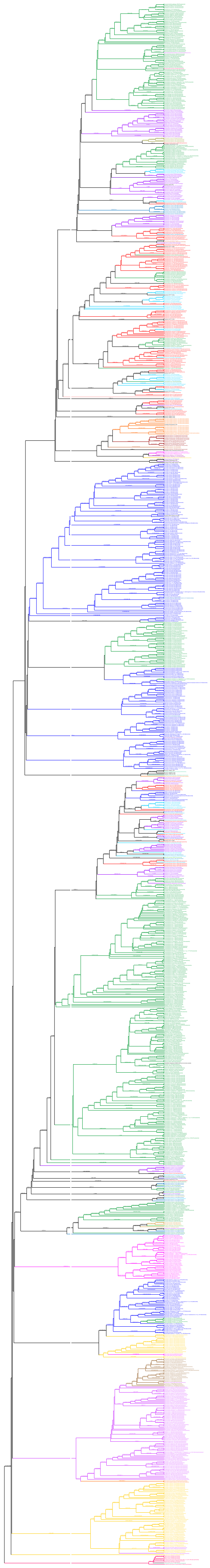

Supplementary Figure S2. The full phylogenetic tree (cladogram) obtained in MrBayes for the HmuY homologs from all phyla. Main bacterial lineages are marked in different colors. The values at nodes indicate in the following order: posterior probabilities found in MrBayes as well as support values calculated by approximate likelihood-ratio test (aLRT) based on a Shimodaira-Hasegawa-like procedure and non-parametric bootstrap calculated both in (more)PhyML and IQ-TREE. The posterior probabilities < 0.5 and the percentages < 50% are omitted or indicated by a dash "-".

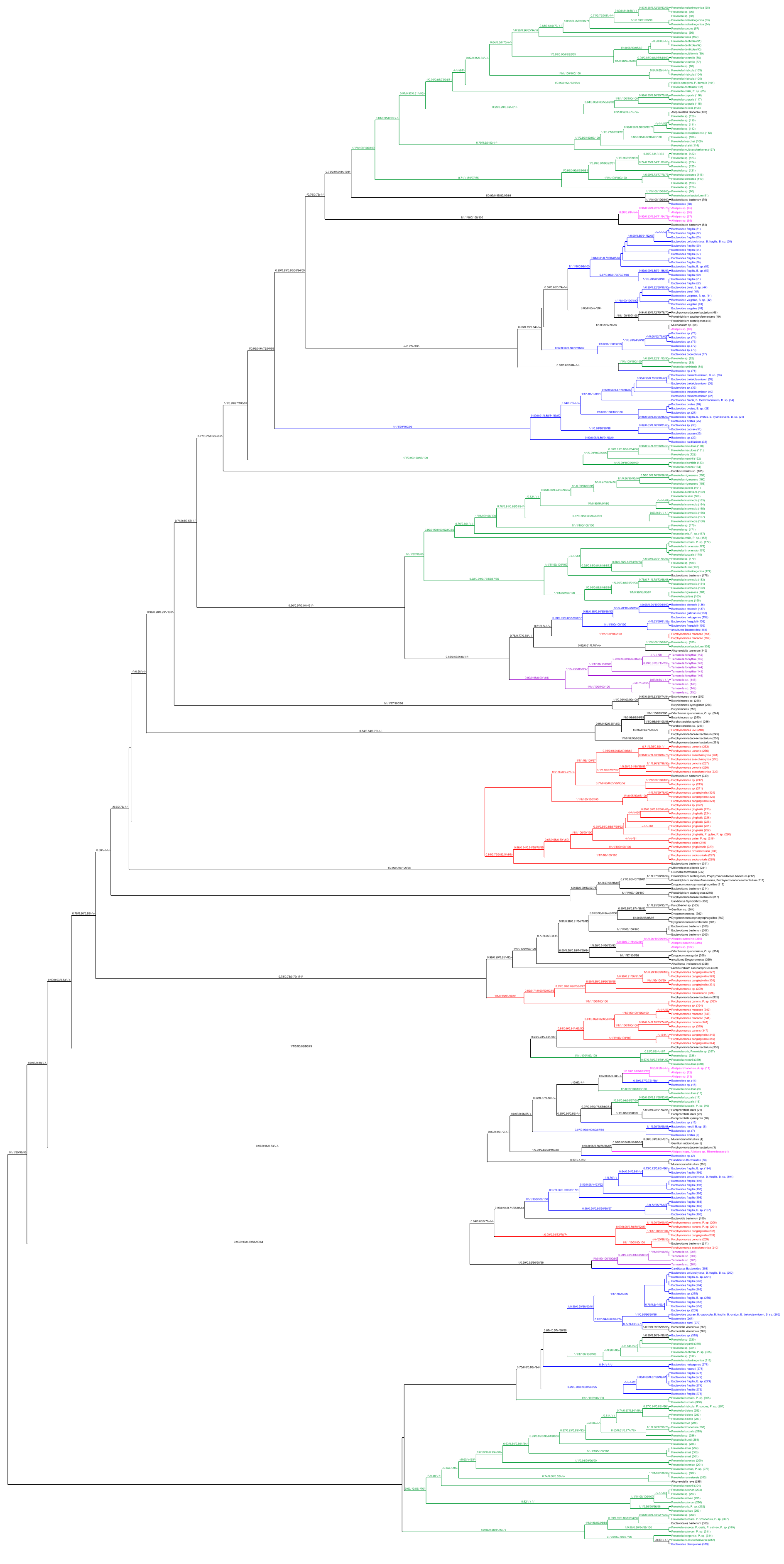

Supplementary Figure S3 The full phylogenetic tree (cladogram) obtained in MrBayes for the HmuY homologs in Bacteroidia. Main Bacteroidia classes are marked in different colors. The values at nodes indicate in the following order: posterior probabilities found in MrBayes and PhyloBayes as well as support values calculated by approximate likelihood-ratio test (aLRT) based on a Shimodaira-Hasegawa-like procedure and non-parametric bootstrap calculated both in (more)PhyML and IQ-TREE. The posterior probabilities < 0.5 and the percentages < 50% are omitted or indicated by a dash "-".

```
HmuR_A7436      ------------------------------------------------MKSVVTKQA---

Tf_HmuR_I       MKSFLGNILFLLLLTVPSGTVFAEDEELEVGRYTVKGTVVDENGDPLPGASVWVVGATIG
Tf_HmuR_II      ---MKRTVTLLFLVLCSMSSLYATEIGAADRDYIIKGTVKNRQDEPIPYATVMVTGTNIG
                                                                  :* .  :

HmuR_A7436      ------------------------LIGLLFFSISIYSHAANPPAQPTDTI-VSGNIALED
Tf_HmuR_I       AGTNANGEFAIRLNEGREYTLRVSFTGYIPEEVKVN---RQTGAQPLHVILKPAENELNE
Tf_HmuR_II      MAANVEGKFQIKLNRG-NYKLRISCTGYEPRTVEVD---AVK-EPELDIVLDEAKNNLTE
                                          *     :.:            . :   .:  * :

HmuR_A7436      IVVTGSRTARLLKDVPVPTKVFKAKDIKAIAPSSFIDVLQYILPGIEFTKHGS---RDQL
Tf_HmuR_I       VVVTGARIARPLKEVPVLTRVISQKDIQALNPMSIETLLQYELPGLQITYN-SMSRMPQI
Tf_HmuR_II      VVVTGTRSEKMLKDEPVVTRVITAEDIKKIDPQDFKSLLEYELPGLHFDGAAHGSGLPAI
                :****:*  : **: ** *:*:. :**: : * .:  :*:* ***:.:           :

HmuR_A7436      NAQGFDESSILFLVDGELIS-TGSTSGIDFERINPDDIERIEVLRGASSALYGSNAIGGV
Tf_HmuR_I       KYQGVDGEYMLFLIDGERVSGEGADHNVDFTRFNVDEIERIEFIRGSQSTVYGSNALGGV
Tf_HmuR_II      SFQGMDSRYLLFLIDGERIAGEGALDNVDFSRLNVDNIERIEIVKSAMSTLYGSNALGGV
                . **.*   :***:*** ::  *:  .:** *:* *:*****.::.: *::*****:***

HmuR_A7436      INIITRTAKDPFRVSASARYDSRDGQKYDVAAGVKRGIFTSQSGVQYRADKSYILADQF-
Tf_HmuR_I       INIITKKANRPFIGNIHARYAGTNGQKYTVSAGTKKDRFSSLTNITFRTRDTYSVGDKNG
Tf_HmuR_II      INIITKKANRPFVGTVSTRLTSRKDRKYTVSLGTRQEKFSSLTTASFSRRDPFTFEERKP
                *****:.*: **  .  :*  . ..:** *: *.::  *:* :   :   . : . ::

HmuR_A7436      -------------------EQELNVAGNTTWNINQKFTISPTENLSFNLTGLVNLRKQHW
Tf_HmuR_I       KTLKIVNPDGTAKDSI-LKAESTTVYGYKVWDASQKFGYAFTDKLSADLRGSFYHNARDI
Tf_HmuR_II      TKRLYELPDGRDSIGVNPKLGKYNMRGYETFSIAQKLGYDFTDKLNAELNGSFYQNKLLY
                                     . .: *  .:.  **:    *::*. :* *  .  .

HmuR_A7436      --TDKIDFLYNSYDVKAGANWRISETSDLDVSYHYDKYSRDTCLIKTENQKKLPIFDEDM
Tf_HmuR_I       PEGGKIQDIFVDYAFNTKVNYVFRDDRRLIVSYNYDNYKKDKDFF-EAKF-KRTDYRNRT
Tf_HmuR_II      VKEGKISERFRDFTFGGKVNYIFNENSRLDLSYHYDNYFKDDVDSADHHK-TKIRYRDIL
                   .**.  : .: .   .*: : :   * :**:**:* :*       :  .   : :

HmuR_A7436      HHLRAQYNLNLAEVHLLNVGLEYIHDNVASPRLSSPNDPGEKSVNNKILYGQYIYKVTPK
Tf_HmuR_I       QMLRADYSGTY-GDHTVSIGSEGFFEYLKHYM---MKDSADARNENYAFYMQEDWKPFSS
Tf_HmuR_II      NNVRLNYSLLLAERHSLTVGSEVNAEKLRTNW---FKDTTANSITNYVLYVQDDYRITDR
                : :* :*.      * :.:* *   : :        :*       *  :* *  ::

HmuR_A7436      LVLSYGGRLDKHSGFGLYYTSRLSAMYKCS-HVTNRLSYAEGYRAPSLQEMYFFFNHGA-
Tf_HmuR_I       LNVIVGVRADYHRKYHWHVTPKVSAMYRPAKTITLRAGYSQGFRSPSLKELYQEYDMGGL
Tf_HmuR_II      LSLVGGIRMDYHSLYKVHVSPKLSAMYKVG-NVTLRGGYAAGFRSPSMKELYGEFSHGG-
                * :  * * * *  :  : : ::****: .  :* * .*: *:*:**::*:*  :. *.

HmuR_A7436      --FFIYGNPDLKPEKSRMLSYSAEAHWNKLTLMGNVFFNHVRNRIDFTY--------KGT
Tf_HmuR_I       GFLMLYGNPDLKPETSHQFSLSAEYTQGGFNASVSTSHNRFHNKITYKE-----KNKNAA
Tf_HmuR_II      -MFKIYGSTDLKPETSHHVSLSGEWTAGIFNISATGYYNWFNKRIAMQQRISENKKQKYP
                  : :**. *****.*: .* *.*   . :.   .  .* ..::*            :

HmuR_A7436      DLIYTNVSKEMRIFGFEGQANVVLPYGFGFRASYSYTYDRWKATDKAGTEMKLSNTRPHA
Tf_HmuR_I       EWQYTNA-EHARTTSVEAIVQMRMDFGLTMTGSYAYVNDYEQVNGK-----NTSSVRPHS
Tf_HmuR_II      DFVYVNA-DSAKTYGADINMQIRLPFDLTLKGTYSYVYDDTKIKGR-----RSSYVRPHT
                :  *.*. .  :  . :   :: : :.: : .:*:*. *  : ..:     . * .***:

HmuR_A7436      ATATLSYGHNFSKNYRLASNFSTRFLSDLKTG---RMNSDELFEEITYPGYTICRLDIEN
Tf_HmuR_I       VTFHAVYSRKFGKI-GTNLALNGQWASQLSTYRFSKDG---THRSVTYDARTLCSLNAGA
Tf_HmuR_II      AVMQAEYSRKFGNC-RATLGLNGRWMSGVDYWATSYVSKKRVFTKLELNDYMIWKLNASC
                ..    *.::*.:       :. :: * :.       .    . .:      :  *:

HmuR_A7436      HFYRNYTLHIGAENLFDYKPKALAFNSP-TSPGRIIYGLVRITF------
Tf_HmuR_I       TMPKGVSVNVGIDNLFNYQDKAADSSLQLPQKGISLIGTVNINLADMFGL
Tf_HmuR_II      RFPKGVTLNIGIDNLFNTKDDNFSDLYAIFSRGTEFVANLSVNIAELIGK
                 : :. ::::* :***: : .         . *  : . : :.:
```

Supplementary Figure S4 Amino-acid sequence alignment of TonB-dependent outer-membrane receptors. Proteins encoded in *P. gingivalis hmu* (HmuR_A7436) and *T. forsythia hmu*-like (Tf_HmuR_I and Tf_HmuR_II) operons are shown. Conserved histidine (H), glutamic acid (E) residues and FRAP, NPNL motifs present in classical TonB-dependent receptors transporting heme are marked in red and blue, respectively.

Porphyromonas gingivalis HmuY

MKKIIFSALCALPLIVSLTSCGKKKDEPNQPSTPEAVTKTVTIDASKYET**WQYFSFSKGEVV**NVTDYKN**DLN**
WDMALHRYDVRLNCGESGKGKG**GAVFS**GKTEMDQATTVPTDGYTVDVLGRITVKYEMGPDGHQMEYEEQGFS
EVITGKKNAQGFASG**GWLE**FSHGPAGPTYKLS**KRVFFVRGADGNIAKVQFTDYQDAE**LKK**GVITFTYTYP**VK

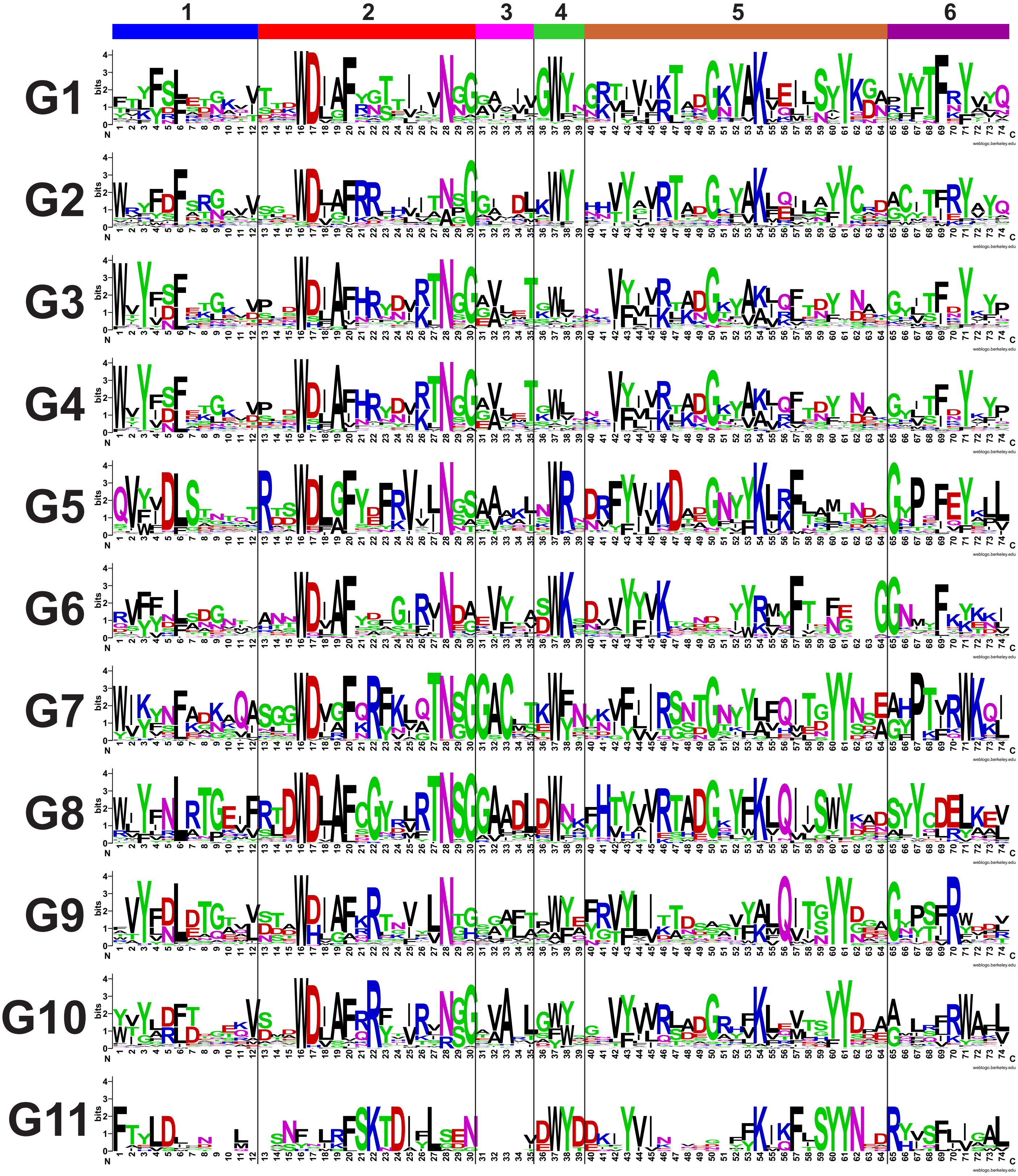

Supplementary Figure S5 Selected sequence regions that were conserved in HmuY homologs. The consensus sequences of eleven main groups indicated in Fig. S1 are presented as sequence logos generated at the http://weblogo.berkeley.edu/logo.cgi. The parts of *P. gingivalis* HmuY sequence corresponding to these conserved regions are marked by different colors and histidine residues binding heme are circled out.

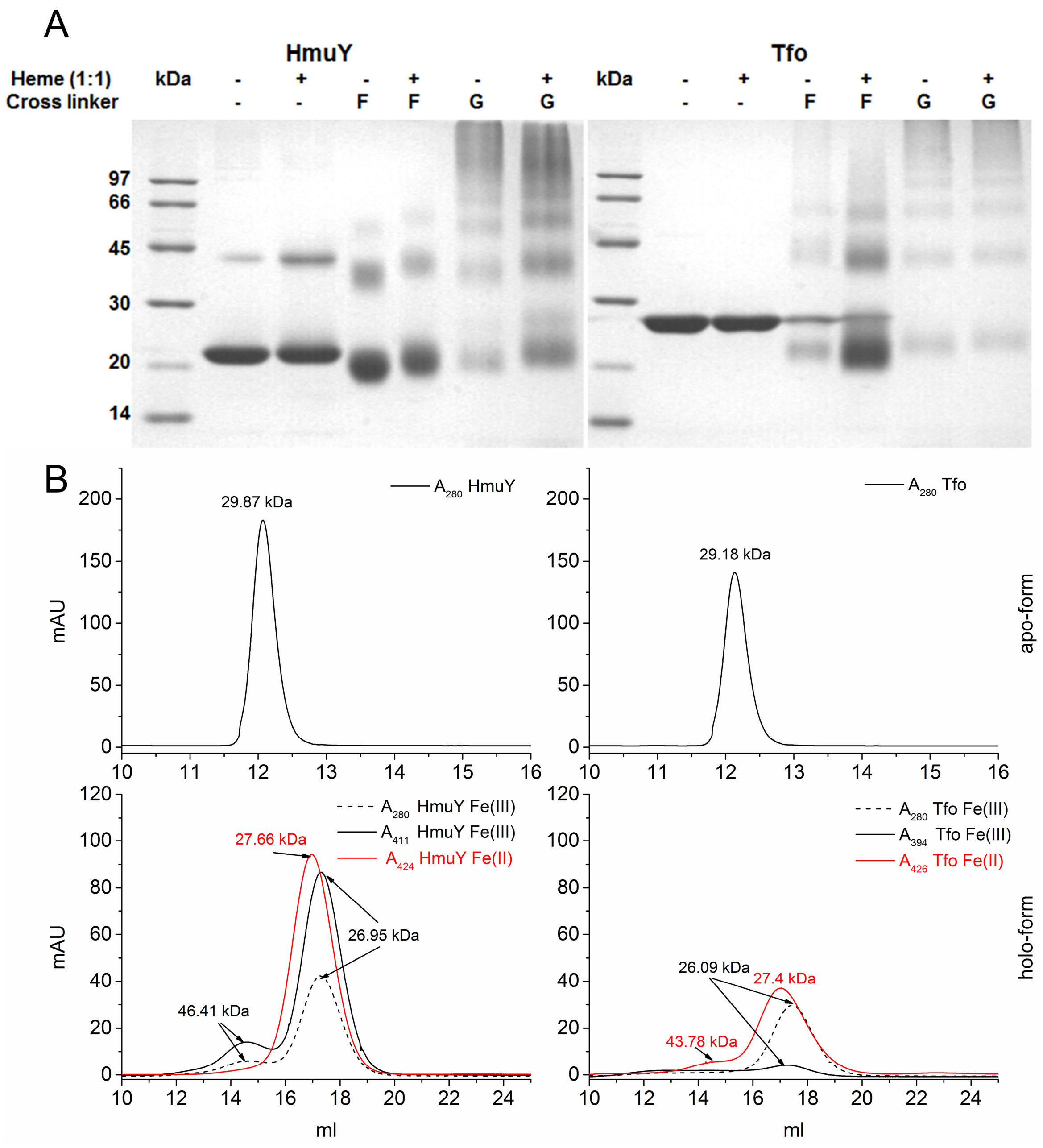

Supplementary Figure S6 Oligomer formation examined by cross-linking analysis and size exclusion chromatography of apo- and holo-forms of *P. gingivalis* HmuY and *T. forsythia* Tfo. **(A)** Cross linking of proteins in apo- and holo-forms was carried out using 1% formaldehyde (F) or 0.1% glutaraldehyde (G) for 1 h at 37°C. **(B)** Analytical size-exclusion chromatography was carried out under air (oxidizing; black solid and dashed lines) and reducing (addition of sodium dithionite; red line) conditions. Samples of HmuY and Tfo (~0.22 mg; 100 µl) in PBS or 200 mM Tris-HCl, containing 140 mM NaCl, pH 8 (under oxidizing or reducing conditions, respectively) were applied in apo- or holo-form onto a Superdex 75 Increase 10/300 GL (GE-Healthcare) or a ProteoSEC 11/30 3-70 HR column (Protein Ark), respectively, connected to AKTA Pure FPLC system (GE Healthcare). To analyze the oligomeric state of proteins under reducing conditions, 30 mM sodium dithionite was added to the separating buffer. Chromatography was carried out with 0.8 ml/min flow rate. Both columns were calibrated using Gel Filtration Markers Kit for Protein Molecular Weights 6,500-66,000 Da (Sigma-Aldrich).

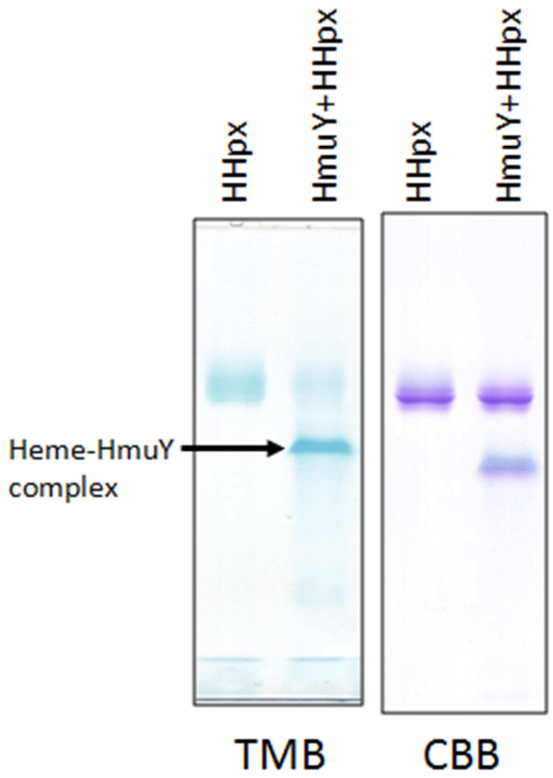

Supplementary Figure 7 Heme extraction from hemopexin-heme complex by *P. gingivalis* HmuY. SDS-PAGE showing formation of the HmuY-heme complex following incubation of apo-HmuY and human hemopexin-heme complex (HHPx) is shown. Gel was stained with tetramethylbenzidine-H_2O_2 (TMB) to reveal the presence of heme and counterstained for proteins with Coomassie Brilliant Blue R-250 (CBB).

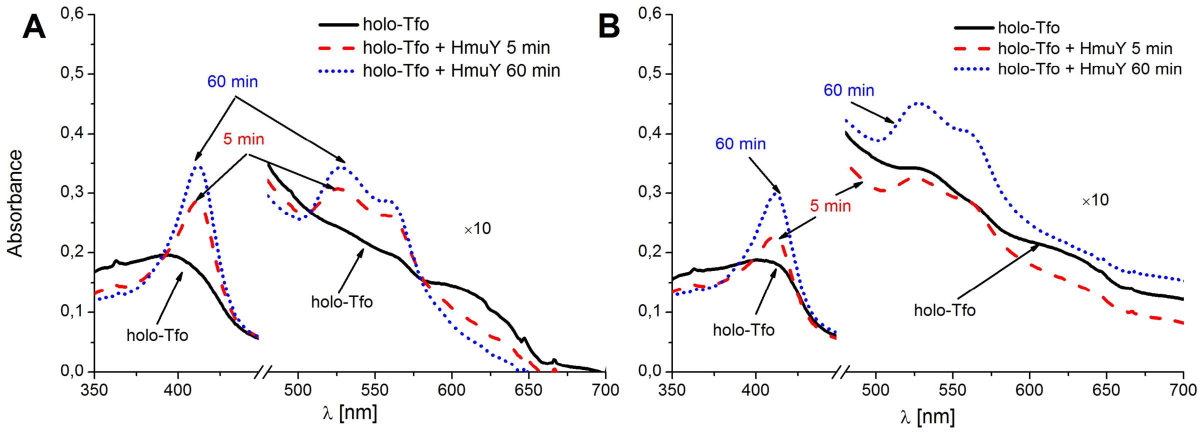

Supplementary Figure S8 Heme sequestration from *T. forsythia* Tfo-Fe(III)heme complex (holo-Tfo) by *P. gingivalis* apo-HmuY. Holo-Tfo (10 μM) was incubated under air (oxidizing) conditions with equimolar concentration of apo-HmuY at pH 7.6 **(A)** or 6 **(B)**. Changes in absorption spectra analyzed by UV-visible spectroscopy are shown at indicated time points.

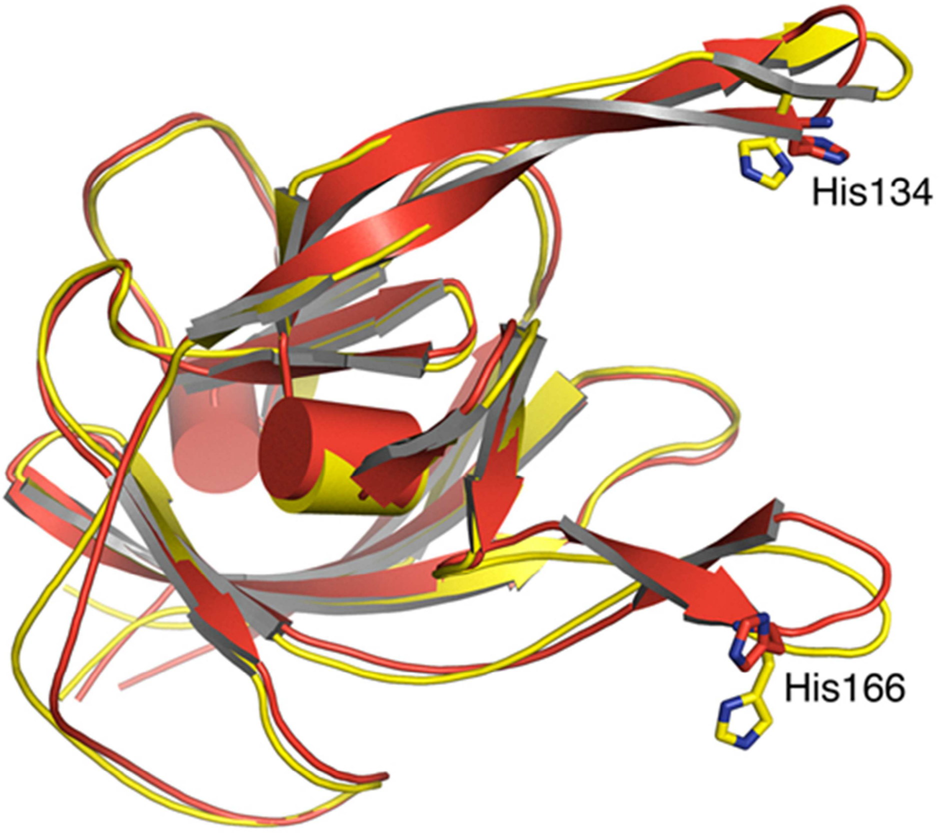

Supplementary Figure S9 Molecular dynamics of HmuY after removal of heme group from 3H8T.PDB. The apo-HmuY crystal structure (yellow) is compared to the molecular simulation (MD) after 8 ns (red). The opening up of the heme-binding pocket by ~16 Å that is observed in the apo-crystal structure (see **Figure 10** in the manuscript), which is due largely to movement of the loop containing His166, is reproduced by the MD.
